# Not sorcery after all: Roles of multiple charged residues in membrane insertion of gasdermin-A3

**DOI:** 10.3389/fcell.2022.958957

**Published:** 2022-09-02

**Authors:** Viktoria Korn, Kristyna Pluhackova

**Affiliations:** Stuttgart Center for Simulation Science, Cluster of Excellence EXC 2075, University of Stuttgart, Stuttgart, Germany

**Keywords:** gasdermin, membrane proteins, free energy, molecular dynamics, computational biology, membrane pore, protein insertion

## Abstract

Gasdermins execute programmatory cell death, known as pyroptosis, by forming medium-sized membrane pores. Recently, the molecular structure of those pores as well as the diversity in their shape and size have been revealed by cryoTEM and atomic force microscopy, respectively. Even though a growth of smaller to larger oligomers and reshaping from slits to rings could be documented, the initiation of the gasdermin pore formation remains a mystery. In one hypothesis, gasdermin monomers insert into membranes before associating into oligomeric pores. In the other hypothesis, gasdermin oligomers preassemble on the membrane surface prior to membrane insertion. Here, by studying the behavior of monomeric membrane-inserted gasdermin-A3 (GSDMA3), we unveil that a monomeric gasdermin prefers the membrane-adsorbed over the membrane-inserted state. Our results thus support the hypothesis of oligomers preassembling on the membrane surface before membrane penetration. At the same time, our simulations of small membrane-inserted arcs of GSDMA3 suggest that the inserting oligomer can be small and does not have to comprise a full ring of approximately 26–30 subunits. Moreover, our simulations have revealed an astonishingly large impact of salt-bridge formation and protein surroundings on the transmembrane passage of charged residues, reducing the energetic cost by up to 53% as compared to their free forms. The here observed free energy barrier of mere 5.6 kcal/mol for the membrane insertion of monomeric GSDMA3 explains the surprising ability of gasdermins to spontaneously self-insert into cellular membranes.

## 1 Introduction

Gasdermins (GSDMs) are a protein family conserved among vertebrates, including mice, rats, horses, and humans, where they play a central role in defense mechanisms eliminating harmful cells through pyroptosis Shi et al. (2017). Pyroptosis is a proinflammatory form of programmed cell death Walle and Lamkanfi (2016) following a range of pathways, either the canonical or non-canonical inflammasome pathway, where the inflammasomes detect cytosolic contamination Kovacs and Miao (2017), or via granzymes. In the final step of pyroptosis, transmembrane lytic pores are formed causing swelling of the cell, followed by the efflux of cytoplasmic content, and cell rupture Zahid et al. (2021). Recently, GSDMs have been shown to be the sole executors of pyroptosis by forming lytic pores Kovacs and Miao (2017).

Before revealing its full necrotic potential, gasdermin lies dormant in a deactivated form consisting of covalently bound C- and N-terminus. Across all variants, the three-dimensional structure is very similar, despite diverging sequences (45% homology Zahid et al. (2021)). Upon inflammation, enzymatic cleavage of gasdermins releases the functional N-terminus of its inhibitory C-terminus, enabling membrane insertion and pore formation. Ding et al. (2016) While human GSDMD can be cleaved by inflammatory caspases 1, 4, 5, and 11 Shi et al. (2015), the specific protease responsible for the cleavage of GSDMA3 remains a mystery.

Gasdermins are involved in a wide range of diseases Liu et al. (2021). GSDMA is associated with and presumably involved in asthma, systemic sclerosis, alopecia, and inflammatory bowel disease Lluis et al. (2011); Yu et al. (2011); Saeki et al. (2009); Terao et al. (2017); Söderman et al. (2015). Also, pyroptosis is surmised to be stimulated in SARS-CoV-2 infections through the non-canonical inflammasome Lee et al. (2020); Zheng et al. (2020). In mice, GSDMA3 is expressed in skin cells and hair follicles Runkel et al. (2004) and is involved in apoptosis Lei et al. (2011) and differentiation Li et al. (2010). Moreover, due to the ability of gasdermins to self-insert into specific membranes independent from insertases and chaperones Ding et al. (2016), GSDMA3 is being researched as a therapeutics specifically disrupting cancer cells and thereby reduce tumor growth Wang et al. (2020).

**FIGURE 1:**
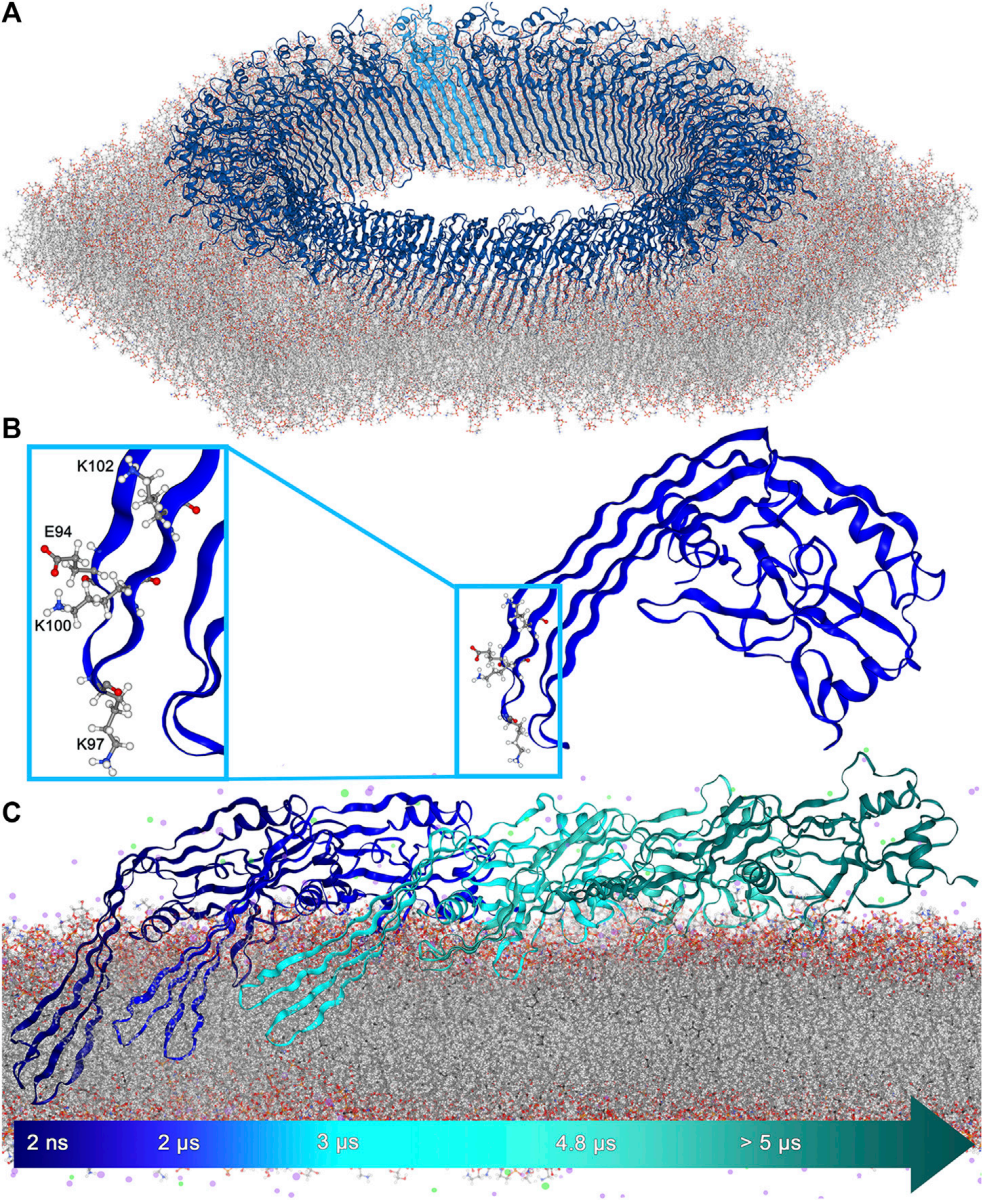
**(A)**. 27-fold GSDMA3 pore in an *E. coli* membrane. GSDMA3 is shown as blue cartoon, with one protomer highlighted in lighter color. The membrane is shown as sticks. Water and ions were omitted for clarity. **(B)**. Detailed representation of the charged amino acids at the tip of GSDMA3’s first β-hairpin. E94 and K100 form a pair due to their opposing charges. **(C)**. The GSDMA3 monomer spontaneously slipping out of the *E. coli* membrane in the course of an atomistic MD simulation with uncharged E94, K97 and K100 (uuu state).

The molecular structure of the gasdermin lytic pore was resolved first in 2018, when Ruan et al. Ruan et al. (2018) published the cryoTEM structure of gasdermin-A3 pores, presenting highly structured β-barrel oligomers consisting of 26–28 monomers, among which the 27-fold pore (Figure 1A) was the most common. Three years later, human gasdermin-D has been shown to preferably form β-barrels comprising 33 protomers Xia et al. (2021). Recently, extensive atomic force microscopy (AFM) investigations have shed light on the growth of gasdermin pores Mulvihill et al. (2018); Mari et al. (2022) and by combining AFM with molecular dynamics (MD) simulations, the opening of the lytic pore by release of the lipids (and proteins) from the center of the oligomer was visualized Mari et al. (2022). Nevertheless, the compelling issue whether gasdermins insert into the membrane and then assemble pores, or whether the membrane-adsorbed oligomers obtained both by AFM and cryoTEM are able to tear into the membrane, remains open.

Usually, membrane proteins insert into the hydrophobic membrane core guided and aided by their hydrophobic domains Ulmschneider et al. (2014); Brambillasca et al. (2005). Yet, an increasing number of membrane proteins have been discovered, which contain multiple polar or even charged amino acid residues in their membrane inserting domains Broecker et al. (2014); Vögele et al. (2019); Altrichter et al. (2017); Brambillasca et al. (2006); van Dalen and de Kruijff (2004); Mulvihill et al. (2015), many of them containing or forming β-barrel pores with hydrophilic residues inside the pore and hydrophobic ones on the outside of the pore where they are in contact with the membrane Bosshart et al. (2012); Vögele et al. (2019); Altrichter et al. (2017); Mulvihill et al. (2015). A non-negligible number of membrane proteins is able to self-insert. Broecker et al. (2014); Vögele et al. (2019); Bosshart et al. (2012) However, as the membrane passage of charged amino acids comes at a very high energetic cost MacCallum et al. (2007); Johansson and Lindahl (2009), insertases like YidC and SecYEG typically aid the process Laskowski et al. (2021); Oswald et al. (2021). The more astounding it is that GSDMA3 self-inserts, as four charged residues, one glutamic acid (E94), and three lysines (K97, K100, and K102) (Figure 1B, Supplementary Figure S2) have to pass, or in case of K102 at least partially insert into the hydrophobic membrane core, during the protein’s insertion process. Other members of the gasdermin family, including the functionally very significant human GSDMD, possess β-hairpins decorated with even more polar and charged residues (Supplementary Figures S3–S5). A couple of mechanisms have been suggested before to aid the insertion of charged amino acids into hydrophobic membrane cores: The formation of water defects where water molecules surround polar and charged residues inside the membrane Ulmschneider (2017); Wang et al. (2014); Allolio et al. (2016); Dorairaj and Allen (2007); Bonhenry et al. (2013); Li et al. (2013), the bending of lipids enabling polar and charged headgroups (e.g. phosphates) to stabilize residues of opposing charge Allolio et al. (2016); Dorairaj and Allen (2007); Li et al. (2013), salt-bridge formation between residues of opposing charges Duart et al. (2022); Mbaye et al. (2019); Chin and von Heijne (2000); Donald et al. (2011), “piggyback” where one charged residue drags water into the membrane and thereby paves the way for membrane insertion of further charged residues at hardly any extra cost MacCallum et al. (2007, 2008), with arginine and its guanidinium cation presenting a special case titled “arginine magic” Vazdar et al. (2018); Allolio et al. (2016); Moon and Fleming (2011), as well as mediating effects of the protein surroundings that provide charge shielding and stabilization Bosshart et al. (2012); Vögele et al. (2019); Altrichter et al. (2017); Moon and Fleming (2011); Cymer et al. (2015); van Dalen and de Kruijff (2004).

To determine the energetic cost of GSDMA3 membrane insertion, we conducted extensive molecular dynamics simulations of its N-terminus with four charge combinations regarding the membrane-passing residues E94, K97 and K100. Our simulations have revealed that the salt-bridge formation between E94 and K100 and the polar protein surroundings of all three charged residues drastically decrease the energy barrier for membrane insertion of GSDMA3’s β-hairpins, in comparison to the insertion cost of individual charged amino acids.

## 2 Methods

The membrane-inserted structure of the N-terminus of murine gasdermin-A3 was based on the recently published cryoTEM structure (rcsb code 6CB8 Ruan et al. (2018)). Modeller 9 Webb and Sali (2016) was used to insert the missing residues 66-PGSS-69 and 234-KIRR-237 to obtain a full pore-forming version of the protein characterized in detail previously Mari et al. (2022). Additionally, two membrane-adsorbed models were prepared. In the first model, termed “pre-inserted”, the β-hairpins (residues F81-L113 and V164-I198) were remodelled as loops by Modeller 9. In the “cleaved” model, the N-terminal half (residues M1-R237) of the X-ray structure 5B5R Ding et al. (2016) of full-length GSDMA3 was extracted and missing residues added by Modeller 9. The monomeric proteins were then inserted into or adsorbed onto an *E. coli* polar lipid extract bilayer Pluhackova and Horner (2021), consisting of 14 different lipids containing 72% phosphatidylethanolamine (PE), 23% phosphatidylglycerol (PG) and 5% cardiolipin lipids with 37% of saturated, 25% cyclopropanylated, and 38% unsaturated lipid tails, using our established multiscaling procedure Pluhackova et al. (2013). First, the simulation system was prepared and equilibrated at coarse-grained resolution, using the Martini3 force field Souza et al. (2021). Then the system was converted to all-atom resolution using backward Wassenaar et al. (2014) and the CHARMM36 forcefield Huang et al. (2017); Klauda et al. (2010) with the TIP4P water model Jorgensen and Madura (1985) as described in the original implementation of the CHARMM force field in GROMACS Bjelkmar et al. (2010) and reequilibrated atomistically. All simulations were performed in GROMACS (2018.x - 2021.x) Abraham et al. (2015). For more details see Supplementary Information, section Supplementary methods.

## 3 Results

The structure of the transmembrane lytic pore formed by GSDMA3 Ruan et al. (2018) (Figure 1A) shows a typical distribution of hydrophobic side chains on the membrane-facing side of the β-barrel and hydrophilic (polar and charged) residues on the water facing surface of the β-barrel Wimley (2002). Following the hypothesis that individual GSDMA3 proteins insert into the membrane and then oligomerize Mulvihill et al. (2018), both the hydrophobic and hydrophilic sides of the monomeric GSDMA3’s β-hairpin face the membrane lipids. Especially the membrane insertion and incorporation of the first β-hairpin is supposed to be energetically unfavorable, as it is decorated by multiple charged residues (Supplementary Figure S2): one lysine in position 102 and two further lysines and one glutamic acid at the tip (E94, K97 and K100) (Figure 1B).

**FIGURE 2:**
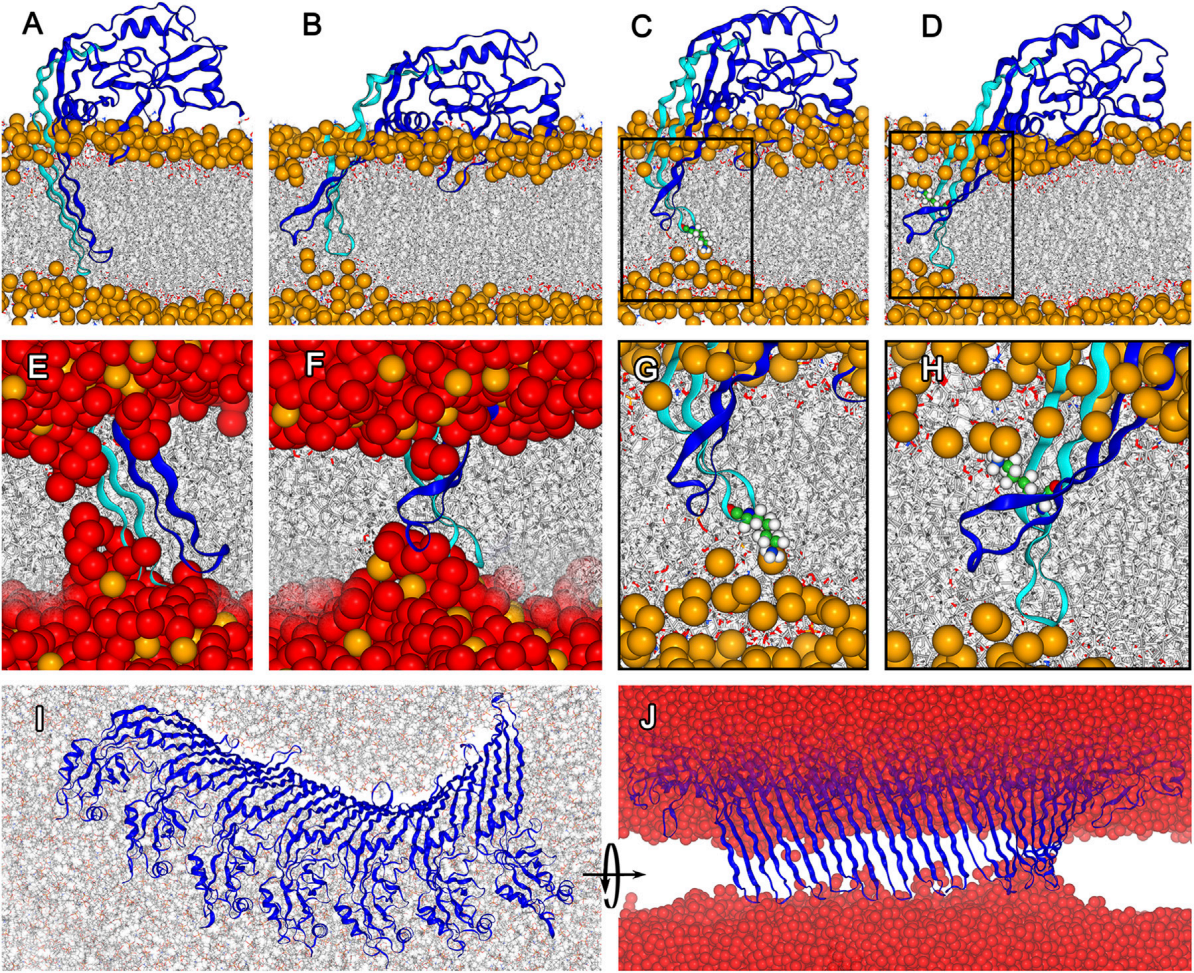
Structural adaptation of membrane inserted GSDMA3 in a monomeric state and forming an arc comprising 7 protomers. **(A)**. Conformation of monomeric GSDMA3 in the beginning of the production run simulations (i.e. at 0 µs). **(B)**. Reshaping of the β-hairpins of monomeric GSDMA3 after 5 μs of simulation time. **(C)**. K97 (highlighted as small spheres) anchors the GSDMA3 monomer to the lipid headgroups in the extracellular membrane leaflet. **(D)**. Snorkeling of K102 (shown as small spheres) into the lipid headgroups of the cytosolic membrane leaflet. **(E)**. Zoom-in on the water defect at 0 μs and **(F)** after 5 µs. **(G)**. Detail view on K97 anchoring from **(C)**. **(H)**. Detail view on K102 snorkeling in **(D)**. **(I)**. Cytosolic view on the deformation of 7-mer arc after 4 μs and **(J)** a detail on the β-hairpins of the 7-mer arc pulling water into the membrane. GSDMA3 is shown as blue cartoon, in **(A)–(H)** the first β-hairpin carrying the charged residues is highlighted in cyan. The membrane is shown as grey sticks with phosphorus atoms highlighted as orange spheres (only in **(A–H)**). Water is shown in **(E), (F)**, and **(J)** as red spheres. Apart from **(I)**, the cytosolic side is found on the top and the extracellular side on the bottom of the membrane. In all here presented simulations, GSDMA3 was in its ccc state (i.e. K97, K100, and E94 were charged).

In order to shed light on the behavior and positioning of the monomeric GSDMA3 adsorbed onto or embedded in a well characterized membrane, where GSDMA3 is known to spontaneously insert into and form oligomers in Mari et al. (2022), we have inserted the GSDMA3 monomer into a model of an *E. coli* polar lipid extract membrane Pluhackova and Horner (2021). This membrane choice is further justified by the facts that GSDMA3 is lethal to bacteria, including *E. coli* Ding et al. (2016) and ruptures mitochondrial membranes of similar lipid composition Rogers et al. (2019). Moreover, in order to unravel the impact of charged residues on the estimated properties, we have varied the protonation state of the three titratable residues at the tip of the first hairpin in our atomistic molecular dynamics simulations (E94, K97, and K100 charged (ccc), all three uncharged (uuu), and E94 and K100 charged but K97 uncharged (cuc)). In the geometric perturbation simulations, the ucu state (E94 and K100 uncharged but K97 charged) was added for comparison purposes. In the membrane-adsorbed state, the orientation of GSDMA3 on the membrane surface stays conserved during the course of the simulations, while the protein diffuses around. The disordered loops, forming β-hairpins in the pore state, sample the conformational space, and temporarily attach to the membrane surface. However, even though a few residues penetrated the membrane/solvent interface, no spontaneous insertion was observed, similarly to recent MD simulations of GSDMD Schaefer and Hummer (2022). Therefore, in the following we concentrate on the behavior of membrane-inserted GSDMA3. During the equilibration phase, the position of the protein in the membrane was restrained to the lytic-pore conformation. This caused water entering the bilayer along the β-hairpins and lipid deformation in the vicinity of the hydrophilic face of the protein (Figures 2A,E). Upon releasing the restraints in the production run simulations the β-hairpins started to reshape and reposition, independently of the protein’s protonation state, in an attempt to minimize the membrane defects (Figures 2B–D). The second β-hairpin, carrying no charged residues, assumed a twisted conformation and left the headgroup region of the extracellular leaflet shifting to the hydrophobic membrane core. This ductile form enabled the monomeric protein to remain membrane-inserted (Supplementary Figures S6-S8) for the simulation time (ranging from 5 to 12.8 μs), except for one uuu simulation, in which the β-hairpins slipped out of the bilayer after 5 µs (Figure 1C, Supplementary Figure S8). Supplementary Figure S9 shows the increased flexibility of the β-hairpins compared to the globular domain by coloring the monomeric GSDMA3 according to the B-factors of the C_*α*_ atoms. In those simulations, as well as in additional simulations of GSDMA3 adsorbed on the membrane surface in a prepore or cleaved state (i.e. with β-hairpins modelled as loops) lasting 5 and 1 µs, respectively, the orientation of the GSDMA3 on the membrane surface and the protein-lipid interactions attaching the globular domain of GSDMA3 to the membrane remained the same as observed in the lytic-pore state Mari et al. (2022); Ruan et al. (2018).

In the membrane-inserted state of monomeric GSDMA3, the first β-hairpin stays deeply inserted and K97, regardless of its charge, anchors the hairpin to the lipid headgroups of the extracellular leaflet causing a membrane dent (Figure 2G). K102 bends to the lipid headgroups of the intracellular membrane leaflet (Figure 2H). Such lysine bending from the hydrophobic membrane core to the polar lipid headgroups has been observed before Pluhackova et al. (2015); Mishra et al. (1994) and is called “lysine snorkeling” Strandberg and Killian (2003). In the simulations with charged E94 and K100 (i.e. fully charged ccc and partially charged cuc states, with K97 being neutral in the latter case) K97 is deeply inserted (average distance between the C_*α*_ of K97 and the membrane center of mass after exclusion of the first 500 ns simulation time for equilibration purposes amounts to −0.60 ± 0.27 nm for ccc and −0.68 ± 0.27 nm for cuc. The error given represents the standard deviation of the distribution and thus the fluctuations of the insertion depth (Supplementary Figures S6, S7). In case of the uuu simulations, both hairpins translate towards the hydrophobic membrane core (−0.27 ± 0.44  nm, Supplementary Figure S8). This positioning of both β-hairpins in the membrane core enabled the β-hairpins to slip out of the membrane in one replica of the uuu simulations, leaving the GSDMA3 monomer adsorbed on the membrane surface. Such behavior of the GSDMA3 monomer made us wonder how the process of membrane excision looks like from an energetic perspective and how the charges of the three amino acids at the tip of one β-hairpin influence the free energy landscape and therefore the membrane passage process.

Using structures from the previous simulations in which the β-hairpins of GSDMA3 are 1) in the transmembrane state, 2) slipping out of the bilayer, and 3) adsorbed on the bilayer surface (Supplementary Table S1) we have performed “umbrella” (geometric perturbation) sampling simulations and constructed the potential of mean force (PMF) describing the excision/insertion of GSDMA3 β-hairpins from/into the membrane, along the reaction coordinate *ζ* represented by the z-distance between the C_*α*_ of K97 and the center of mass of the bilayer. Thus, we could avoid including structures and conformations from far-from-equilibrium steered molecular dynamics simulations, which pose often a problem in terms of convergence and accuracy of PMF estimation Blazhynska et al. (2022). However, one has to keep in mind that even though the above mentioned structures overlap over the whole span of our reaction coordinate, we can not assure to be sampling the complete conformational space exhaustively. Nevertheless, the convergence of our PMFs and the comparably small error bars support the following statements. The PMFs revealed two local minima (Figures 4I,J, Supplementary Figure S10). The minimum at *ζ* ≈ 2 nm corresponds to the membrane-adsorbed state of the β-hairpins. In this state, many polar and charged residues (mainly K97) form energetically favorable interactions with intracellular lipid headgroups (Supplementary Figures S23–S25). The second minimum, at *ζ* ≈ −0.25 nm, corresponds to the transmembrane state of the β-hairpins of GSDMA3. In this state, K97 is interacting with the lipid headgroups in the extracellular membrane leaflet (Supplementary Figure S23). A maximal energy barrier between these two minima, corresponding to the cost of the insertion/excision, amounted to 5.57 kcal/mol in the ccc simulation. While this barrier agrees perfectly with the experimental value of the free energy required for membrane insertion of lysine in context of a transmembrane β-barell OmpLA amounting to 5.39 ± 0.52 kcal/mol Moon and Fleming (2011), it contrasts with the energy barrier that a single charged lysine (10.15 kcal/mol), or charged lysine analog (7.46 kcal/mol) experiences when passing the bilayer (Figure 4K, Supplementary Figures S15, S16, S21, S22). These values are smaller than those obtained previously by MacCallum et al., who have predicted a barrier of almost 15 kcal/mol for a charged lysine side chain analog (described by the OPLS-AA force field Kaminski et al. (2001); Rizzo and Jorgensen (1999)) passing the DOPC bilayer (described by the Berger force field Berger et al. (1997)) MacCallum et al. (2007). Similarly, Sandoval-Perez and Pluhackova et al. have observed an energy barrier of 14 kcal/mol for a charged lysine residue including a backbone capped with acetyl on the N-terminus and an amine group on the C-terminus passing a POPC bilayer (all described by the CHARMM36 force field) Sandoval-Perez et al. (2017). Our values of 10.15 and 7.46 kcal/mol thus suggest that 1) the negatively charged *E. coli* PLE bilayer makes an insertion of a positively charged lysine molecule a little more favorable than the zwitterionic POPC bilayer, 2) self-insertion of positively charged sole lysine into this membrane is highly improbable under physiological conditions, and 3) the protein surroundings of the lysine drastically reduce its free energy insertion barrier.

**FIGURE 3:**
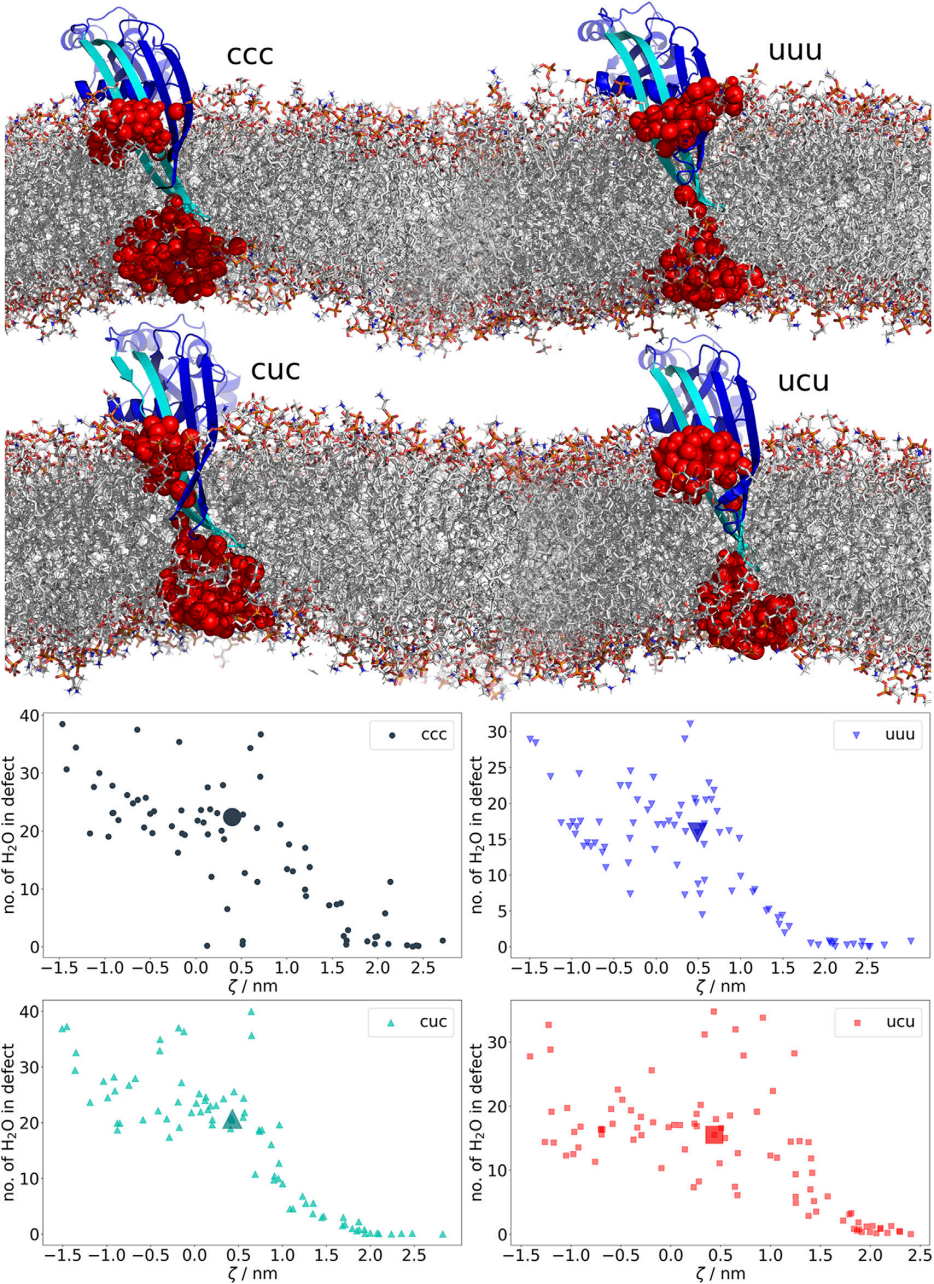
Water-filled membrane defects caused by β-hairpins of GSDMA3. The plots at the bottom show the average number of water molecules in the defect in each trajectory from the geometric perturbation. Coloring of the datapoints is based on the charges of E94, K97, and K100 (u = uncharged, c = charged). As soon as GSDMA3 comes into contact with the membrane at *ζ* ≈ 2, water-filled membrane defects arise. The highlighted large datapoints correspond to the visualized structures above. In the molecular representations, water oxygens in membrane defects are shown as red spheres. GSDMA3 is visualized as blue cartoon, with the first β-hairpin highlighted in cyan. The membrane is shown as sticks.

Visual investigation of the trajectories gave first hints on differences in membrane deformations and water defects regarding the β-hairpin versus the lysine-only passage through the bilayer. Due to the too high dewetting cost, the charged lysine pulls 18.5 and the analog 7.9 water molecules into the bilayer (averaged over −1 ≦ *ζ* ≦ 1) and exposes them to the hydrophobic membrane core (Supplementary Figures S26, S27), which is known to be energetically costly Wang et al. (2014). In Figure 3, the even larger defects formed by GSDMA3 regardless of its charge state are shown. When the protein is fully inserted, it pulls in water molecules from both intra- and extracellular surroundings. Thereby the polar surface of the β-hairpins accommodates on average 18.5, 17.0, 13.0, and 14.0 water molecules in the ccc, cuc, ucu, and uuu states, respectively, in the hydrophobic center of the membrane (−1 ≦ *ζ* ≦ 1). Very rarely a single ion briefly diffused into the water defects inside the hydrophobic membrane core.

**FIGURE 4:**
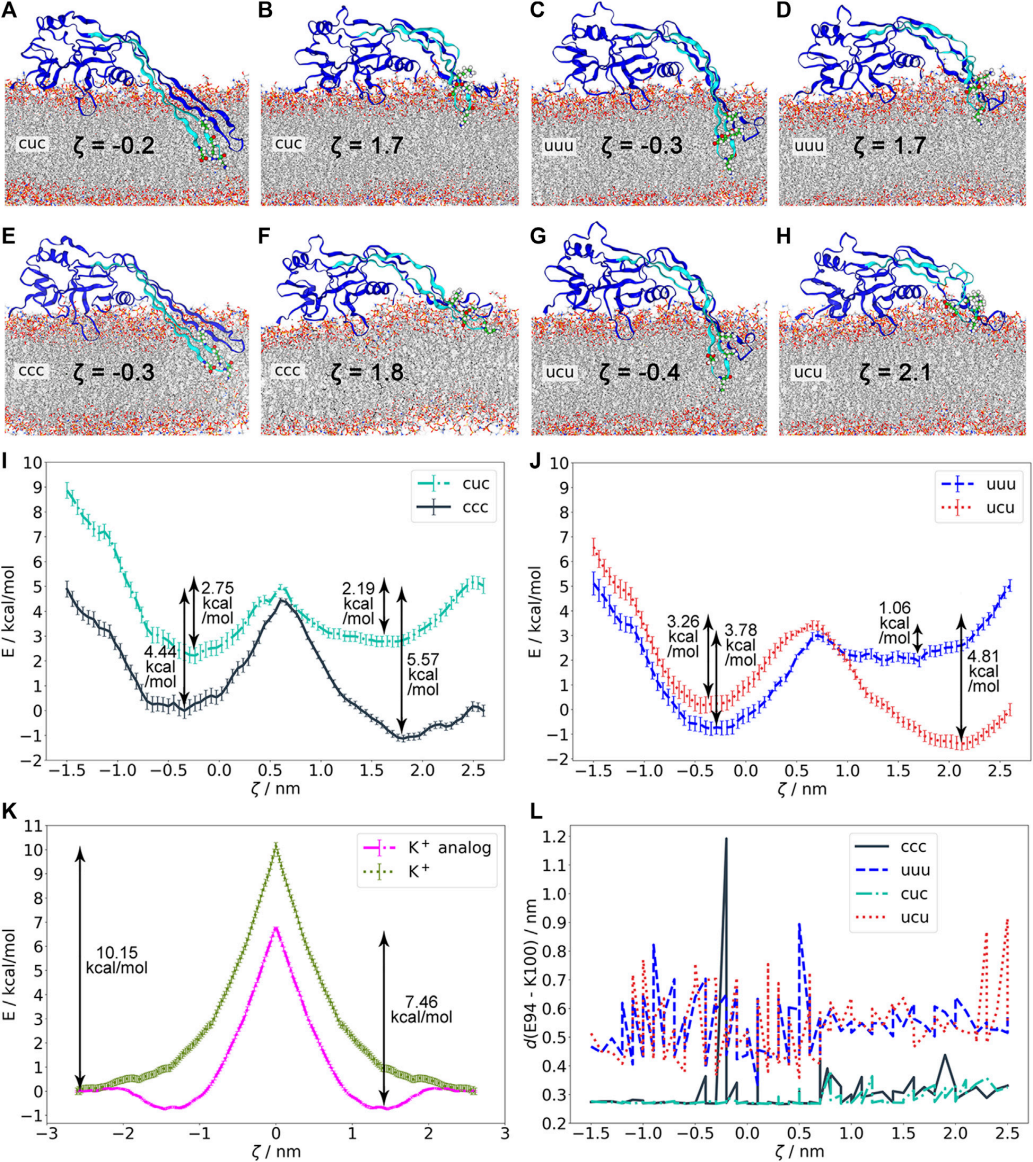
Energetics of membrane insertion/excision of GSDMA3 β-hairpins as studied by geometric perturbation simulations in various charge states of GSDMA3. **(A)–(H)**. Protein structures and residues E94, K97, and K100 in the minima of the potentials of mean force (PMFs) shown in **(I)–(J)**. **(I)**. PMFs of ccc and cuc. **(J)**. PMFs of uuu and ucu. The cuc and uuu curves have been shifted at *ζ* = 2.5 nm relative to the ccc and ucu, respectively, by the free energy required to deprotonate a lysine residue in bulk water at pH 7.0 (5.03 kcal/mol). With K97 charged, the membrane-adsorbed state is preferred (ccc, ucu). If E94 and K100 are neutral, K97 is also deprotonated inside the hydrophobic membrane core. **(K)**. PMFs of lysine^+^ and the lysine^+^ analog entry into the *E. coli* membrane showing that the individual amino acids have higher free energy barriers upon entering the membrane than the GSDMA3 monomer. **(L)**. Distance between the residues E94 and K100 in various charge states and in different membrane insertion depths defined by the reaction coordinate *ζ*. If charged, E94 and K100 form a salt bridge. The reaction coordinate *ζ* in **(I)–(J)** is defined as the distance between the C_*α*_ of K97 and the center of mass of the membrane. The error bars behind the PMF curves denote standard deviations estimated over 100 bootstraps.

However, in the case of GSDMA3 not only one lysine but four charged residues and a number of polar residues (Supplementary Figure S2) have to insert into the bilayer for the β-hairpin to reach the membrane-inserted state. Two of those residues, E94 and K100, often formed a salt-bridge in our simulations (Figure 4L), therefore they were charge-neutralized pairwise. The PMF curves show that the charge of the E94-K100 pair has only a marginal impact on the height of the energy barrier and on the position and depth of the two energy minima. Figure 4L shows that the deeper these two residues are inserted in the membrane, the closer they stay together. In their charged state, E94 and K100 form a salt bridge (indicated by a distance shorter than 0.4 nm), but even in their neutral form, polar interactions keep E94 and K100 close together. Such close contact isolates the (partial) charges from the surroundings, effectively shielding them from the hydrophobic membrane core. Moreover, it can be supposed that the interaction between E94 and K100 contributes to the stability of the β-hairpin. Comparing the PMFs with varying charge on K97 (Figures 4I, J) reveals that the charge of this residue mainly determines the relative depth of the two minima and thus the preference of GSDMA3’s β-hairpin for the membrane-adsorbed or the membrane-inserted state: If K97 is charged (ccc, ucu), the membrane-adsorbed state is preferred over the transmembrane state and vice-versa. Shifting the PMF curve in the cuc (relative to ccc) and uuu (relative to ucu) states at maximum *ζ* (where K97 is found in the solvent) by the free energy required to deprotonate a lysine residue in bulk water at pH 7.0 MacCallum et al. (2008); Johansson and Lindahl (2009), allows us to estimate whether the K97 uncharged state is more favorable at some insertion depth than the charged state, which is prevalent in aqueous solution (Figure 4). As the curves of cuc and ccc never cross and the latter state is lower in energy in solution, the ccc state is preferred over the cuc state even in the membrane hydrophobic core, despite its higher insertion barrier (5.57 kcal/mol vs. 2.19 kcal/mol). Considering the shapes of the PMFs for uuu and ucu (Figure 4J), it is evident that the uuu curve shows hardly any barrier (1.06 kcal/mol) within the membrane core and has a deeper minimum (3.78 kcal/mol) than the ucu curve (3.26 kcal/mol) in the membrane inserted state. Interestingly, after shifting the uuu curve relative to the ucu PMF curve in solution by the deprotonation free energy of K97 (5.03 kcal/mol), it appears that contrary to the cuc state, K97 prefers to be neutralized if inserted deep in the hydrophobic membrane core. Obviously, synergistic effects between the E94-K100 pair and K97 exist. Yet, the uuu and ucu states are energetically much less favorable than the ccc and cuc states (Supplementary Figure S10) and thus do not represent physiologically relevant states of membrane inserted GSDMA3.

Salt bridge formation leads to even more extreme pKa values of the involved amino acids, i.e. the pKa of acids is reduced and that of bases increased Yang et al. (1993). The more the pKa deviates from the pH of interest (here 7), the larger is the neutralization free energy which is defined as Δ*G* ≈ ± (2.303*RT*) ⋅ (pKa–pH). Therefore, the sum of neutralization free energies of individual K97 (Δ*G* = 4.35 kcal/mol Johansson and Lindahl (2009)), E94 (Δ*G* = 4.02 kcal/mol Johansson and Lindahl (2009)), and K100 (Δ*G* = 4.35 kcal/mol Johansson and Lindahl (2009)), i.e. 12.39 kcal/mol, represents the lowest boundary of the free energy difference between the ccc and uuu PMF curves in solution. Thus, neither the ucu nor the uuu PMF curve will ever cross the ccc PMF curve, which obviously represents the most stable state. Therefore, our simulations show that even though individual amino acids are expected to neutralize in the hydrophobic core of the membrane MacCallum et al. (2007); Sandoval-Perez et al. (2017), amino acids which are part of a complex protein do not necessarily have to do so. Concludingly, our findings suggest that neither K97, nor K100 and E94 neutralize upon membrane insertion of GSDMA3 β-hairpins and monomeric GSDMA3 favors the membrane-adsorbed over the membrane-inserted state. In order to support this hypothesis, we have investigated in detail our recent simulations of arcs consisting of seven GSDMA3 protomers in the *E. coli* PLE membrane Mari et al. (2022). The simulations lasting 2.5 and 4 μs show that even though the overall shape of the arc changes slightly, with the C-terminal part of the oligomer twisting pronouncedly (Figure 2I), the transmembrane orientation of the GSDMA3 protomers mostly resembles the conformation in the cryoTEM pore. However, the protomers at the edges, especially at the C-terminal end where the second hairpin is not stabilized by H-bonds to the neighboring chain, leave the rigid pore conformation. This adaptation is clearly indicated by small root mean square deviations (RMSD) of the hairpins relative to the cryoTEM structure in the center of the arc and increasing RMSDs at the ends of the arc (Supplementary Figure S28). Moreover, the C-terminal end exhibits larger RMSDs. More detailed analysis revealed that both the β-sheets and the hairpin tips contribute, albeit the tips’ RMSD is always larger in agreement with their higher structural plasticity. In the beginning of the simulations, when the GSDMA3 protomers were position restrained to the conformation in the cryoTEM pore, water defects were present along the whole arc. After 4 μs the tips and the side chains at the polar side of the hairpins reoriented so that the only water defect is present at the twisted C-terminal end of the arc (Supplementary Figures S29–S32).

All our findings taken together suggest that GSDMA3 pre-assembles pores on top of a lipid membrane prior membrane insertion, during which all titratable residues on the GSDMA3’s β-hairpins retain their charged state.

## 4 Discussion

Recently, cryoTEM Ruan et al. (2018), AFM experiments Mari et al. (2022) and multiscaling MD simulations Mari et al. (2022) have shed light on the shape and growth of GSDMA3 oligomers and their pre-pore to pore transition by (lipid) unplugging. Nevertheless, because the membrane insertion of gasdermins succeeds without a vertical collapse of the protein, which is characteristic e.g. for pneumolysin van Pee et al. (2016), perfringolysin O Czajkowsky et al. (2004), or suilysin Leung et al. (2014), the initial step of GSDMA3 pore formation consisting of oligomerisation and membrane insertion and their timely order remained hidden. Here, our extensive all-atom MD simulations of the GSDMA3 monomer and the energetics of its membrane insertion have revealed that the pore-forming N-terminus of GSDMA3 prefers the membrane-adsorbed over the membrane-inserted state if the protein is in its monomeric form. Such membrane-adsorbed GSDMA3 monomers explain the small dots observed in time-lapse AFM in our recent study Mari et al. (2022), which are present only in case of both the protease TEV and GSDMA3 in solution without rinsing. As these dots do not appear in controls containing either full-length GSDMA3 lacking the protease or the protease only, they must be comprised by cleaved GSDMA3. Also their disappearance after rinsing by a buffer solution, which also removes membrane adsorbed pre-pore rings from the membrane surface, indicates that the dots capture adsorbed instead of inserted GSDMA3 monomers or small oligomers. These findings support the hypothesis that GSDMA3 oligomers preassemble on the membrane surface prior to membrane insertion and pore formation. The required minimal size and preferred shape of those oligomers for their spontaneous membrane insertion as well as molecular details on the insertion pathway remain to be unraveled.

At the same time, our simulations have unveiled interesting aspects of the membrane passage of charged amino acids in the protein context. Membrane insertion of protein-attached charged amino acids appears to have completely different energetics than the membrane passage of individual amino acids or their side chain analogs. This is true for both single charged amino acids as well as their pairs. Astonishingly, the charge of the amino acid pair E94 and K100 does neither affect the preferred minimum of the protein on or in the membrane, nor does it significantly impact the energy barrier for membrane insertion/excision of GSDMA3’s β-hairpins. These observations hint to the fact that salt bridge formation of nearby amino acids together with their protective protein surroundings shield the charged residues from the hydrophobic membrane core, thus helping GSDMA3 to insert and settle in the membrane. A recent publication has shown that a salt bridge between a charged lysine and glutamic acid is most stable under intermediate microhydration, as it can be found in water-solvated protein cavities Pluhařová et al. (2012), or in our case, in water-filled membrane defects. Also, the energy barrier for the membrane passage of K97, localized at the tip of the first β-hairpin, appears to be significantly reduced (5.57 and 4.81 kcal/mol if K97 is positively charged) compared to the membrane insertion of a charged lysine (10.15 kcal/mol) or a charged lysine analog (7.46 kcal/mol) alone. Thereby, the energy barrier of the membrane insertion of GSDMA3’s β-hairpins in their completely charged state (5.57 kcal/mol) agrees astonishingly well with the energy cost of membrane insertion of a lysine residue located centrally on a transmembrane β-barell of OmpLA (5.39 kcal/mol) Moon and Fleming (2011). Even smaller energy barriers for membrane passage were observed for simulations with deprotonated K97 (i.e. cuc and uuu states). Our extensive equilibrium simulations allowed us to even observe a spontaneous passage of the β-hairpins in the uuu state over the bilayer. Even though the protein thus switched to an energetically less favorable membrane-adsorbed state (in case of the uuu charge state) the observation of this rare event goes hand in hand with the comparably low energy barrier for membrane insertion of GSDMA3. We thus hypothesise that the insertion capability of GSDMA3 is likely to be modulated by molecules stabilizing the membrane-inserted or the membrane-adsorbed state of the protein, e.g. membrane composition or transmembrane proteins. As mentioned elsewhere, the discrepancies between the calculated and experimental insertion free energies of individual charged amino acids or charged amino acids in a protein context result from different microscopic environments rather than from methodological errors Gumbart and Roux (2012). The high energy barrier for the membrane transition of charged amino acids is typically accounted to the formation of water-filled defects reaching into the hydrophobic membrane core Ulmschneider (2017); Wang et al. (2014); Allolio et al. (2016); Dorairaj and Allen (2007); Bonhenry et al. (2013); Li et al. (2013). Here, we observe similar water defects upon the membrane insertion of GSDMA3’s β-hairpins. Obviously, the polar and charged amino acids on the hydrophilic face of the hairpins stabilize the water inside the membrane, thus lowering the energy barrier for the membrane passage of charged amino acids similarly to the mechanism used by the passive membrane insertase YidC Chen et al. (2022). In summary, protein surroundings significantly aid the membrane insertion of charged amino acids by 1) providing a microhydrating environment around charged residues through stabilizing water-filled membrane defects, 2) enabling pair formation between oppositely charged residues, and 3) shielding charged residues from the hydrophobic membrane core. As polar and charged residues cooperate upon membrane insertion in a “piggyback” MacCallum et al. (2007) manner rather than contributing individually to the energy barrier, such nonadditivity poses a challenge for the applicability of hydrophobicity scales predicting membrane-insertion energies of transmembrane proteins.

## Data Availability

The raw data supporting the conclusions of this article will be made available by the authors, without undue reservation.
