# Supplementary material for: Not sorcery after all: Roles of multiple charged residues in membrane insertion of gasdermin-A3
Source: Front Cell Dev Biol. 2022 Sep 2;10:958957. doi: 10.3389/fcell.2022.958957 (PMC9479151; doi:10.3389/fcell.2022.958957)
Supplement: Supplementary file 1 [file DataSheet1.pdf]

Supplementary Material: Not sorcery after all: Roles of multiple charged residues in membrane insertion of gasdermin-A3

Viktoria Korn and Kristyna Pluhackova

1 SUPPLEMENTARY METHODS

Modeller 9 Webb and Sali (2016) was used to insert missing residues 66-PGSS-69 and 234-KIRR-237 to the cryoTEM structure of the membrane-inserted N-terminus of murine gasdermin-A3 (rcsb code 6CB8 Ruan et al. (2018)) as well as to inser missing residues P66-S80 and 170-GNANAIFSL-178 and 188-GSLNNN-193 to the X-ray structure of solvated inactive full length gasdermin-A3 (rcsb code 5B5R Ding et al. (2016)). In the latter structure the residues V238-R453 were deleted to obtained the "cleaved" model of N-terminal murine GSDMA3. The "preinserted" model was prepared by remodelling the β-hairpins (residues F81-L113 and V164-I198) in the membrane-inserted model based on the cryoTEM structure 6CB8 as loops by Modeller Webb and Sali (2016). The three models are visualized in Figure S1.

The protein was energy minimized in vacuo and then coarse-grained into Martini3 force field Souza et al. (2021) using martinize2 (kindly obtained from Siewert-Jan Marrink). The *E. coli* polar lipid extract membrane Pluhackova and Horner (2021), water and ions (100 mM NaCl) were added using insane Wassenaar et al. (2015a) and the simulation system was energy minimized and equilibrated (using 2 fs timestep for 100 ps, then 5 fs timestep for 50 ps, 10 fs timestep for 1 ns, and 20 fs timestep for 1 ns with a restrained protein and in the last equilibration simulation with restrained protein backbone only). The Martini simulations (20 fs timestep, 1 µs) were run using the v-rescale thermostat at 310 K and $\tau_{\mathrm{T}} = 1$ ps. The pressure was controlled at 1 bar using the Berendsen barostat Berendsen et al. (1984) and $\tau_{\mathrm{p}} = 12$ ps, treating the system semiisotropically with a compressibility of $3 \cdot 10^{-4}$ bar^{-1}. Close-ranging (within 1.1 nm De Jong et al. (2016)) electrostatics were described by the reaction field and $\epsilon_{\mathrm{r}} = 15$. Van-der-Waals interactions were cut off at the same distance De Jong et al. (2016) using the potential-shift-Verlet van-der-Waals modifier. To maintain its secondary structure, both the β-sheets and the globular domain of GSDMA3 were stabilized individually using RubberBands Wassenaar et al. (2015b) ($p = 3$, $a = 1$, force constant = 50 kJ/(mol·nm), distance = 1.5 nm). Backward Wassenaar et al. (2014) was used to convert the equilibrated coarse-grained structures to all-atom resolution of the CHARMM36 forcefield Huang et al. (2017)Klauda et al. (2010) and the TIP4P water model Jorgensen and Madura (1985). The protein structure was then refitted by the energy minimized cryo-TEM structure and the surroundings energy minimized before minimizing the entire system. Equilibration was first run for 10 ps at a timestep of 0.002 ps with the protein restrained. Temperature and pressure were controlled by the Berendsen thermostat and barostat Berendsen et al. (1984) to remain at 310 K ($\tau_{\mathrm{T}} = 0.5$ ps) and 1 bar ($\tau_{\mathrm{p}} = 5$ ps). The compressibility was set to $4.5 \cdot 10^{-5}$ bar $^{-1}$ for the semiisotropic system. All hydrogens were equilibrated for 20 ns using a 0.002 ps timestep followed by an equilibration (20 ns, 0.002 ps timestep) with the protein backbone restrained. The production simulations were run for three different protonation states (E94, K97, and K100 charged (ccc), all three uncharged (uuu), and E94 and K100 charged but K97 uncharged (cuc)). To keep the temperature at 310 K, the Nosé-Hoover thermostat Evans and Holian (1985) was coupled by $\tau_{\mathrm{T}} = 0.5$ ps and the Parrinello-Rahman barostat Parrinello and Rahman (1980,

1981) with $\tau_{\mathrm{p}} = 1$ ps kept the pressure of the semiisotropic system (compressibility $= 4.5 \cdot 10^{-5}$ bar $^{-1}$) at 1 bar. Hydrogen atom vibrations were constrained by the LINCS algorithm Hess (2008), far-reaching electrostatics interactions (beyond 1.2 nm) were modelled using particle-mesh-Ewald Darden et al. (1993) and the potential-shift-Verlet modifier while van-der-Waals interactions between 0.8 and 1.2 nm were shifted to zero by the potential-switch method. The buffer tolerance was set to 0.005 kJ/(mol·ps·atom) and the neighbor list updated every 0.02 ps by the Verlet cut-off scheme Páll and Hess (2013). The production simulations were run for five to 12.8 µs (all individual runtimes are listed in the Supplementary Table S1). In all all-atom simulations the integration timestep of 0.002 ps was used. All simulations were carried out in GROMACS (2018.x-2021.x) Abraham et al. (2015). In simulations of monomeric GSDMA3, placed in a hexagonal box of $13.5 \times 11.7 \times 13.5$ nm one copy of GSDMA3 was surrounded by 491 *E. coli* polar lipid extract (PLE) lipids Pluhackova and Horner (2021), about 50 000 water molecules and neutralized by 171 Na^{+} and 30 Cl^{-} ions. In simulations of 7-arc GSDMA3, seven copied of GSDMA3 were placed into an *E. coli* PLE bilayer comprising 1344 lipids, solvated by about 100 000 water molecules and 383 Na^{+}, resulting in a hexagonal box of $23.0 \times 19.9 \times 11.0$ nm.

Table S1. List of all atomistic simulations.

Simulation type	Protein type	Charge state	Length	Sum
MD	monomer inserted	uuu	8.8 µs, 12.3 µs, 5.8 µs[a,b]	26.9 µs
	monomer inserted	ccc	5 µs, 5µs	10 µs
	monomer adsorbed	ccc	5µs[c], 1 µs[d], 1 µs[e]	7 µs
	monomer inserted	cuc	5 µs[a], 3 µs	8 µs
umbrellas	monomer	ccc	76 x 50 ns	3.8 µs
	monomer	cuc	82 x 50 ns	4.1 µs
	monomer	ucu	90 x 50 ns	4.5 µs
	monomer	uuu	85 x 50 ns	4.25 µs
MD	7-mer arc[f]	ccc	2.5 µs, 4 µs	6.5 µs
umbrellas	K^{+}	charged	49 x 50 ns	2.45 µs
	K^{+} analog	charged	43 x 50 ns	2.15 µs

[a] initiated from a 5µs ccc,
[b] β-hairpins slipped spontanously out of the bilayer,
[c] "preinserted" model, i.e. residue 81-113 and 164-198 were remodelled in the cryoTEM structure Ruan et al. (2018) by Modeller Webb and Sali (2016) as loops,
[d] started from 5µs of the uuu simulation in which the β-hairpins slipped out of the bilayer (described in [b]),
[e] "cleaved" model, i.e. missing residues in the N-terminus of the full GSDMA3 from the crystal structure 5B5R Ding et al. (2016) were modelled as loops by Modeller Webb and Sali (2016),
[f] simulations were performed within the framework of our previous publication Mari et al. (2022).
Total simulation time 80 µs.

1.1 Potential of Mean Force Simulations

The frames used for the "umbrella" geometric perturbation sampling of GSDMA3 were extracted from a 5.8 µs long simulation in which gasdermin-A3 (uuu) spontaneously slipped out of the membrane.

Additional structures in the membrane-inserted state were taken from a 5 μs equilibrium simulation in the ccc state, and further conformations on the membrane surface stem from a 1 μs equilibrium ccc simulation. the overlap of the structures from these simulations along the reaction coordinate is visualized in Figure S10. The same GSDMA3 umbrella conformations were used for each charge state (ccc, uuu, cuc, and ucu) by adaptation of the protonation state and the number of Na^+ and Cl^- ions, so that the system was always neutral and contained the same number of atoms for each state. The charge adaptation was followed by energy minimization. As a reaction coordinate ζ, the position of the C_α of K97 relative to the center of mass (COM) of the membrane were chosen. The maximal spacing between the windows along ζ amounted to 0.05 nm. Each of the windows per charge state (see Table S1) was sampled for 50 ns, yielding a total simulation time of more than 3 μs with a timestep of 2 fs. A constraining potential of 500 or 1000 kJ was applied to each window to achieve the best possible overlap of the windows. At a temperature of 310 K, the Nosé-Hoover thermostat Evans and Holian (1985) with $\tau_\mathrm{T} = 0.5$ was utilized along with a pressure of 1 bar and the semiisotropic pressure coupling with the Parrinello-Rahman barostat Parrinello and Rahman (1980, 1981) and $\tau_\mathrm{p} = 5.0$. Intermolecular forces were simulated using a vdW-shift between 0.8 and 1.2 Å and PME electrostatics (0.12 Å grid) with a coulomb radius of 1.2 Å. The potentials of mean force (PMFs) were extracted using the Weighted Histogram Analysis Method (WHAM) Kumar et al. (1992); Souaille and Roux (2001); Hub et al. (2010) implemented in GROMACS. The error bars were obtained from 100 bootstraps.

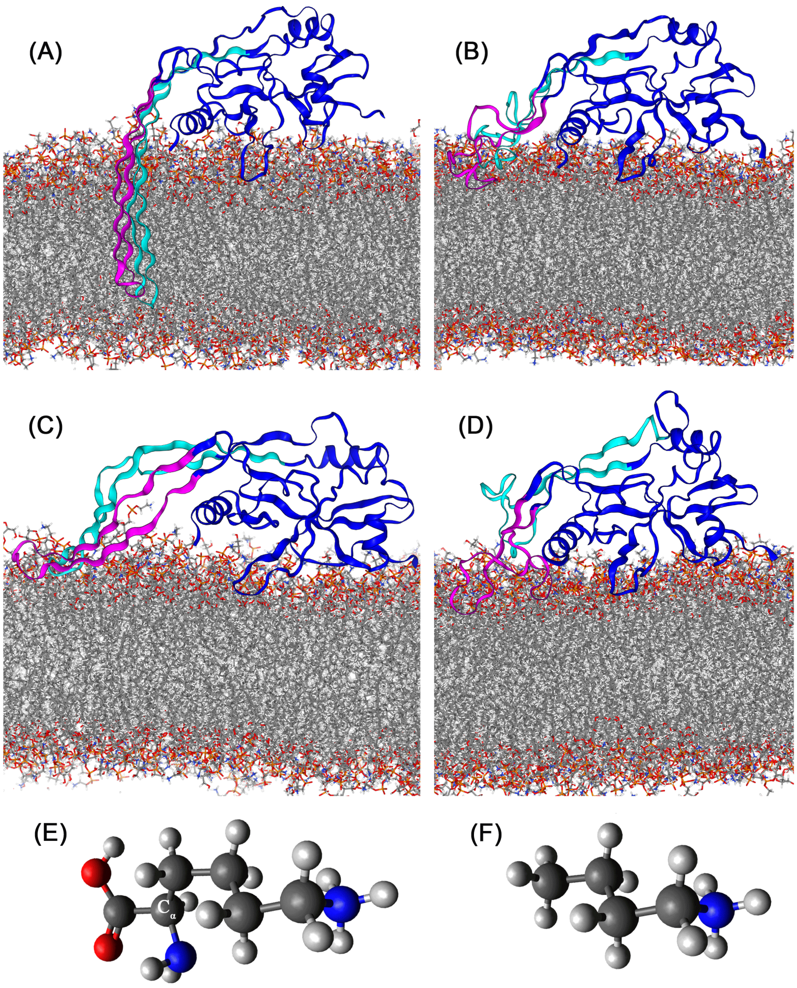

Figure S1. Starting structures of the equilibrium simulations: **(A)** GSDMA3 in its pore conformation, **(B)** ”preinserted” model (see Table S1), **(C)** started from 5 μs of the uuu simulation in which the β-hairpins slipped out of the bilayer, **(D)** ”cleaved” model (see Table S1). GSDMA3 is shown as blue cartoon with the β-hairpins highlighted in cyan and pink, respectively. Molecular representation of **(E)** lysine, and **(F)** lysine side chain analog (n-butylamine).

Lysine and a lysine side chain analog (n-butylamine) were placed into the solvent in 0.6 nm distance to the *E. coli* PLE membrane in a preequilibrated rectangular simulation box spanning $11.1 \times 11.1 \times 7.8\,\text{nm}$ and containing 386 lipids, 16365 water molecules, and a neutralizing NaCl concentration. The water molecules overlapping with the amino acid (analog) were deleted and the system was energy minimized and equilibrated for 10 ps with a timestep of 0.001 ps at a temperature of 310 K controlled by the v-rescale thermostat ($\tau_\text{T} = 0.5$ ps) and a pressure of 1 bar controlled by the Berendsen barostat Berendsen et al. (1984) ($\tau_\text{p} = 5.0$ ps) in a semiisotropic manner with a compressibility of $4.5 \cdot 10^{-5}$ bar^{-1}. Hydrogen bonds were restrained using the LINCS algorithm Hess (2008), electrostatics beyond 1.2 nm was modelled by particle-mesh Ewald Darden et al. (1993) and van-der-Waals interactions were shifted to zero between 0.8 and 1.2 nm by the potential-switch method. To obtain frames for geometric perturbation sampling, the pull code implemented in GROMACS was used to pull the aminoacid through the membrane in z-direction. 49 windows between 2.5 nm and -1.5 nm distance of the C_α from the membrane's center of mass were

selected for lysine. For its analog, 43 windows were selected. An NpT equilibration was run for 10 ns in each window using a timestep of 0.002 ps. All equilibrations were kept at a temperature of 310 K by the Berendsen thermostat Berendsen et al. (1984) ($\tau_T = 0.5$ ps) and coupled semiisotropically to the Berendsen barostat Berendsen et al. (1984) at 1 bar and $\tau_p = 1.0$ ps. The compressibility was set to $4.5 \cdot 10^{-5}$ bar^{-1}, hydrogen bonds were constrained using the LINCS algorithm Hess (2008). Neighbor search was cut off by the Verlet method, far-reaching electrostatics were modelled by particle-mesh Ewald Darden et al. (1993) beyond 1.2 nm. The actual geometric perturbation simulations were run for 50 ns using a timestep of 0.002 ps at a temperature of 310 K controlled by the Nosé-Hoover thermostat Evans and Holian (1985) ($\tau_T = 0.5$ ps) and coupled semiisotropically to the Parrinello-Rahman barostat Parrinello and Rahman (1980, 1981) at 1 bar and $\tau_p = 5.0$ ps at a compressibility of $4.5 \cdot 10^{-5}$ bar^{-1}. Hydrogen bonds were restrained using the LINCS algorithm Hess (2008), electrostatics beyond 1.2 nm were modelled by particle-mesh Ewald Darden et al. (1993) and van-der-Waals interactions were shifted to zero between 0.8 and 1.2 nm by the potential-switch method. The shifts of the PMF curves were calculated using $\Delta G \approx \pm(2.303RT) \cdot (\mathrm{pKa} - \mathrm{pH})$, $\mathrm{pKa(lysine)} = 10.53$ at 311.15 K O'Neil (2006), and $\mathrm{pKa(glutamic\ acid)} = 4.25$ Hunt (2022).

1.2 Analysis

1.2.1 Water defects

The water defect analysis in the bilayers was perfomed using Python 3.x Python (2020) and MDAnalysis Gowers et al. (2019); Michaud-Agrawal et al. (2011). The number of water molecules entering the bilayer due to the presence of the protein was estimated by selecting water molecules residing within the membrane (bordered by the z-coordinates of the phosphate headgroups in each leaflet, determined in each frame individually) and within a cylinder (height 20 Å, radius 10 Å) around the center of geometry of the β-hairpins (residues 92-102 and 176-186). For lysine, a cylinder around its center of geometry with a height of 14 Å and a radius of 10 Å was used, for the analog, a height of 10 Å was chosen.

1.2.2 Residue-lipid headgroup contacts

To find out whether the lysines 97, 100, and 102 were snorkeling into the headgroup region of the membrane, contacts (i.e. distance smaller than 5.5 Å) between the lysines' N atom of its NH_2 or NH_3^+ group and phosphate groups' oxygen atoms were counted in each frame.

1.3 Situation at $\zeta = 2.5$ nm

To ensure that shifting the PMF curves at $\zeta = 2.5$ nm by the experimental free energy cost of (de)protonating in bulk water is valid, the titratable residues have to be surrounded by water so that they can be (de)protonated. Therefore we have measured the distance between the titratable residues and the membrane at $\zeta = 2.5$ nm, and in case of the membrane-near K97 we have estimated the number of water molecules in the first solvation shell of the aminogroup, i.e. within 4 Å of the amino hydrogens. According to the results summarized in Table S2, the residues E94 and K100 are found far away from the bilayer. K97 is well hydrated and despite its membrane proximity, allowing its (de)protonation.

Table S2. Solvation of the amino group of K97 (estimated as the average water molecules that surround the hydrogens of K97's -NH_3^+/-NH_2 group within a 4 Å radius) and the distances between the titratable groups of residues K97, E94, and K100 and the membrane surface (represented by phosphate oxygens) at $\zeta = 2.5$ nm.

Charge state	No. of H_2O around HZ*(K97) within 4 Å	d(OE(E94)-OP*) / Å	d(HZ*(K97)-OP*) / Å	d(HZ*(K100)-OP*) / Å
ccc	4	12.3	3.4	12.3
cuc	3	12.2	3.0	11.6
ucu	4	15.5	4.9	13.2
uuu	3	13.1	3.2	10.2

2 SUPPLEMENTARY FIGURES

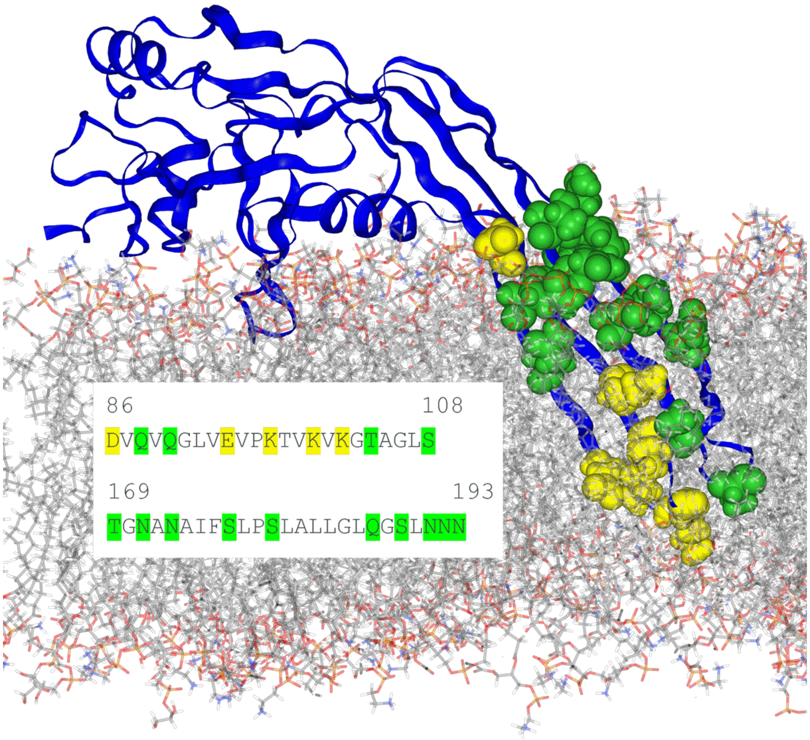

Figure S2. GSDMA3, simulated cryoTEM conformation Ruan et al. (2018), shown as blue cartoon inside an *E. coli* membrane. Polar amino acids are highlighted in green and charged amino acids in yellow. Inset: Sequence of GSDMA3's β-hairpins.

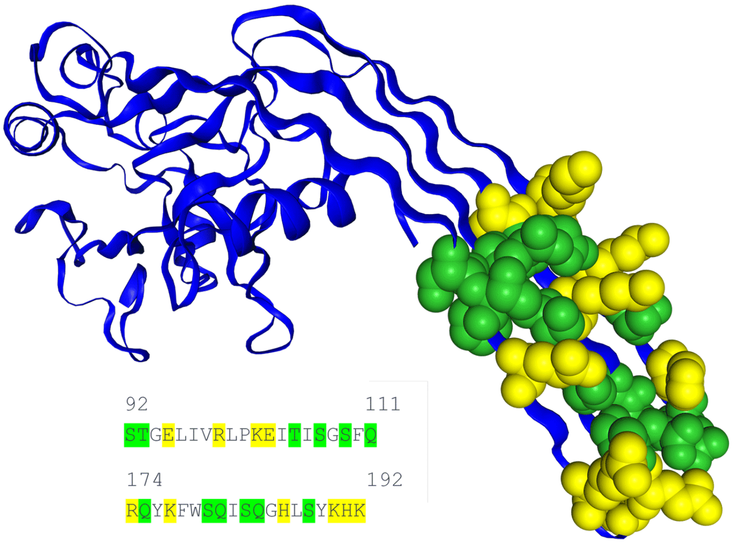

Figure S3. GSDMB (human, modelled onto GSDMA3 with Swiss-Model Waterhouse et al. (2018); Bienert et al. (2017); Guex et al. (2009); Studer et al. (2020)) shown as blue cartoon. Polar amino acids are highlighted in green and charged amino acids in yellow. Inset: Sequence of GSDMB's β-hairpins.

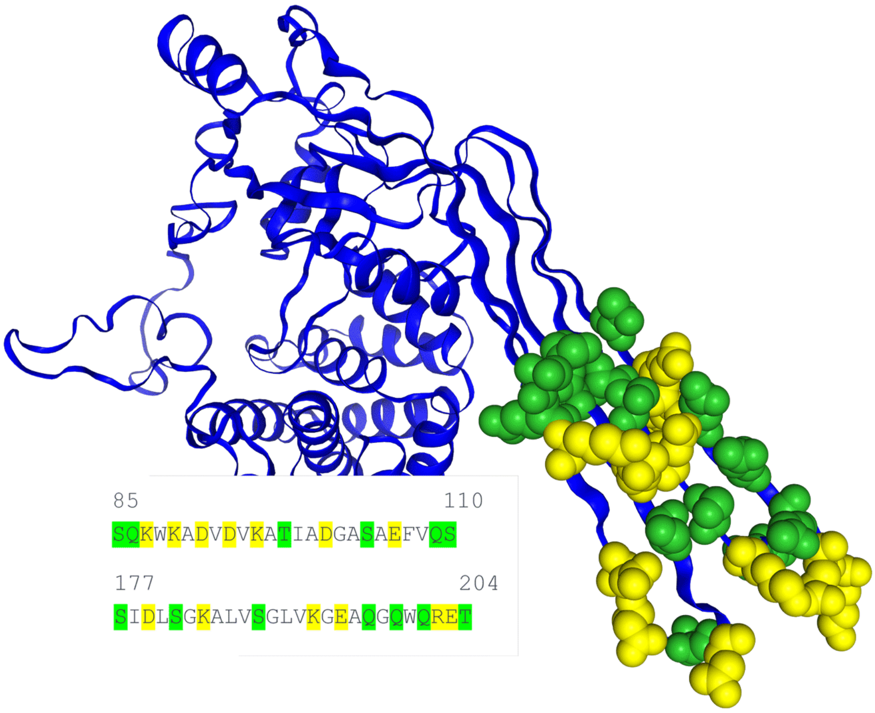

Figure S4. GSDMC (rat, AlphaFold model AlphaFold (2022)) shown as blue cartoon. Polar amino acids are highlighted in green and charged amino acids in yellow. Inset: Sequence of GSDMC's β-hairpins.

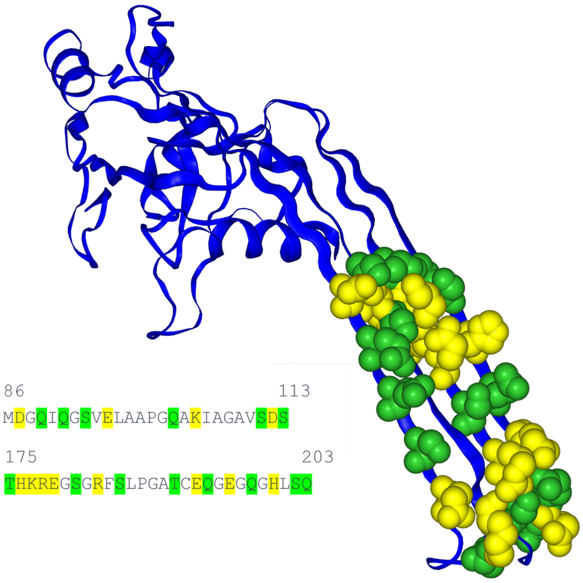

Figure S5. GSDMD (human, cryoTEM structure Xia et al. (2021)) shown as blue cartoon. Polar amino acids are highlighted in green and charged amino acids in yellow. Inset: Sequence of GSDMD's β-hairpins.

2.1 Positioning of K97's C_α relative to the membrane's center of mass in the equilibrium simulations

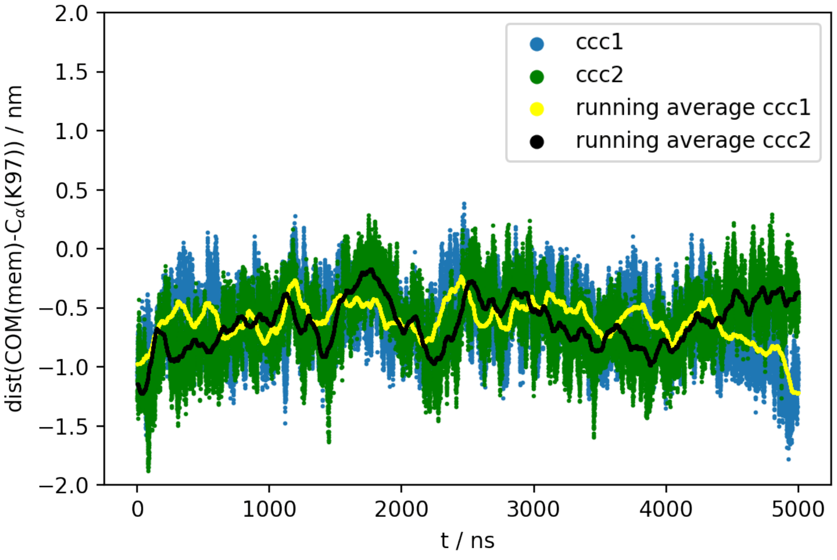

Figure S6. Fluctuating distance between K97's C_α and the membrane's center of mass (ccc).

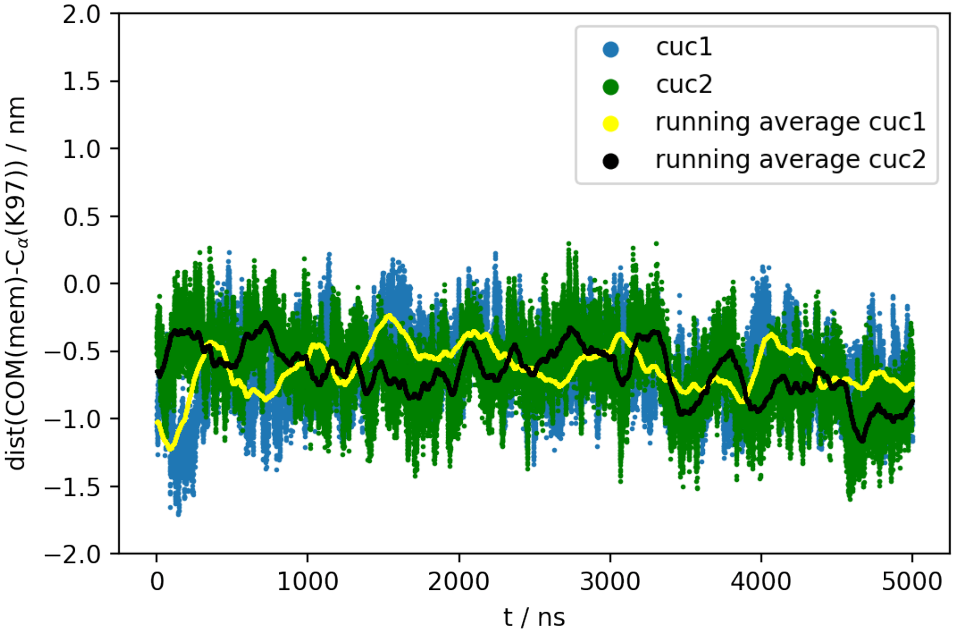

Figure S7. Fluctuating distance between K97's C_α and the membrane's center of mass (cuc).

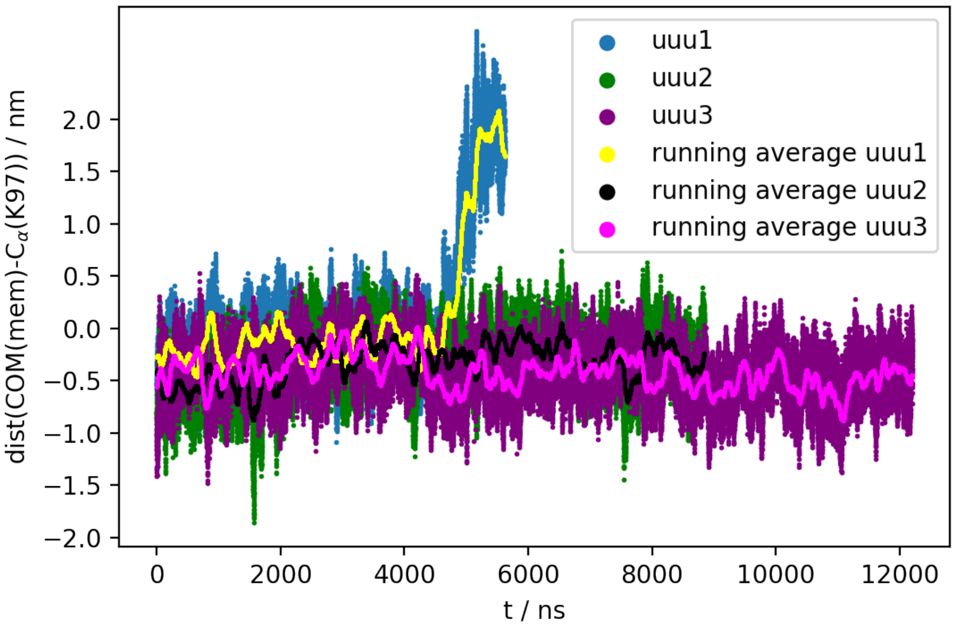

Figure S8. Fluctuating distance between K97's C_α and the membrane's center of mass (uuu). At the end of the uuu1 simulation, the β-hairpins slip out of the membrane.

2.2 B-factors of the GSDMA3 monomer in the equilibrium simulations

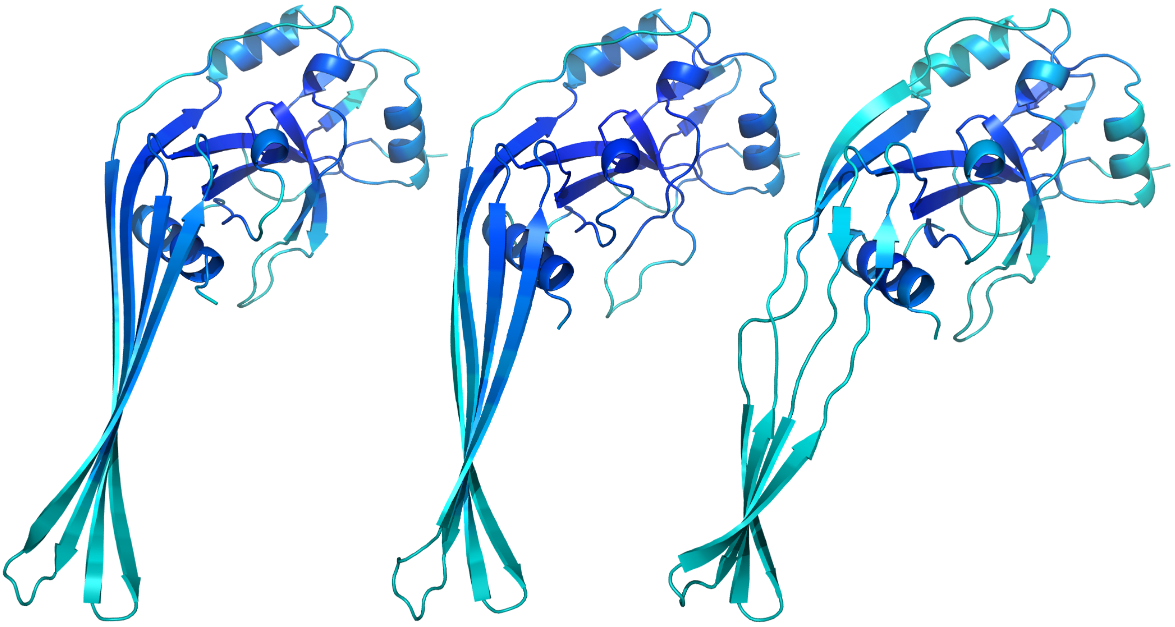

Figure S9. GSDMA3 colored by B-factor (0-200, blue to teal). The blue parts move the least over the course of the simulation, the teal parts show the most movement. Left: ccc2 (average structure). Center: cuc (average structure over cuc 5-10 μs). Right: uuu1 (average structure).

2.3 PMFs

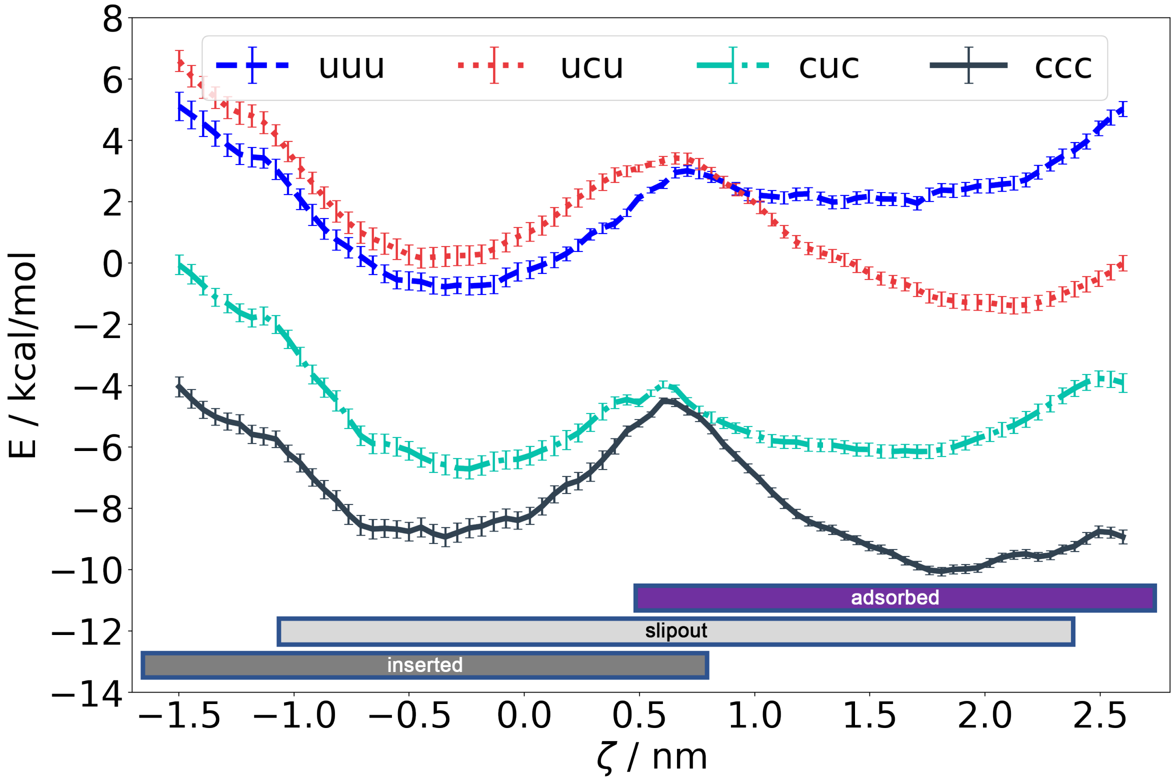

Figure S10. PMFs of four different charge states of the GSDMA3 monomer relative to the ucu state. uuu has been shifted at $\zeta = 2.5$ nm by 5.03 kcal/mol (the free energy cost of deprotonating lysine in bulk water), cuc by -3.90 kcal/mol (the free energy gain of deprotonating glutamic acid in bulk water), and ccc by -5.03 kcal/mol and -3.90 kcal/mol (free energy gains of protonation of a lysine and deprotonation of a glutamic acid in bulk water, respectively). The PMFs of the GSDMA3 monomer with the charged pair E94 and K100 (cuc and ccc) lay lower in energy and do not intersect with the ones of the uncharged pair (uuu and ucu). Therefore, E94 is highly unlikely to be protonated and K100 to be deprotonated. ccc is found lowest in energy, along the whole reaction coordinate, however, around the energy barrier at $\zeta \approx 0.7$ nm the cuc and ccc states are found very close in energy. Thus spontaneous protonation change of K97 in this position can't be excluded. The error bars show standard deviations estimated over 100 bootstraps. The bars at the bottom visualize the span of the reaction coordinate covered by structures extracted from the following simulations: purple: 1 µs equilibrium ccc simulation in the membrane-adsorbed state, light grey: a 5.8 µs long simulation in which gasdermin-A3 (uuu) spontaneously slipped out of the membrane, and dark grey: 5 µs equilibrium simulation of the membrane-inserted conformation in the ccc state.

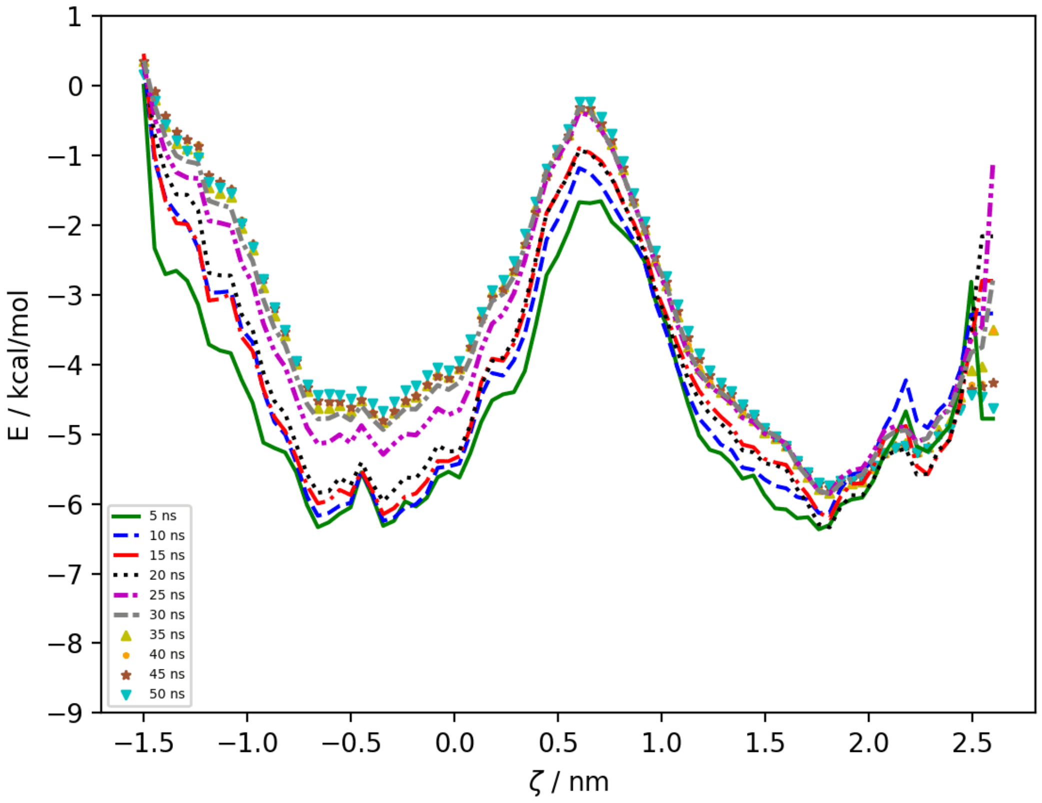

Figure S11. Convergence of the PMF for ccc estimated over increasing simulation times (5 ns equals 0-5 ns of the individual simulations, 50 ns equals the entire simulation time of 50 ns of all individual trajectories). The curves were optimally aligned by calculating their root mean sqare deviation from each other in Python: $\sqrt{(PMF1 - PMF2)^2}$

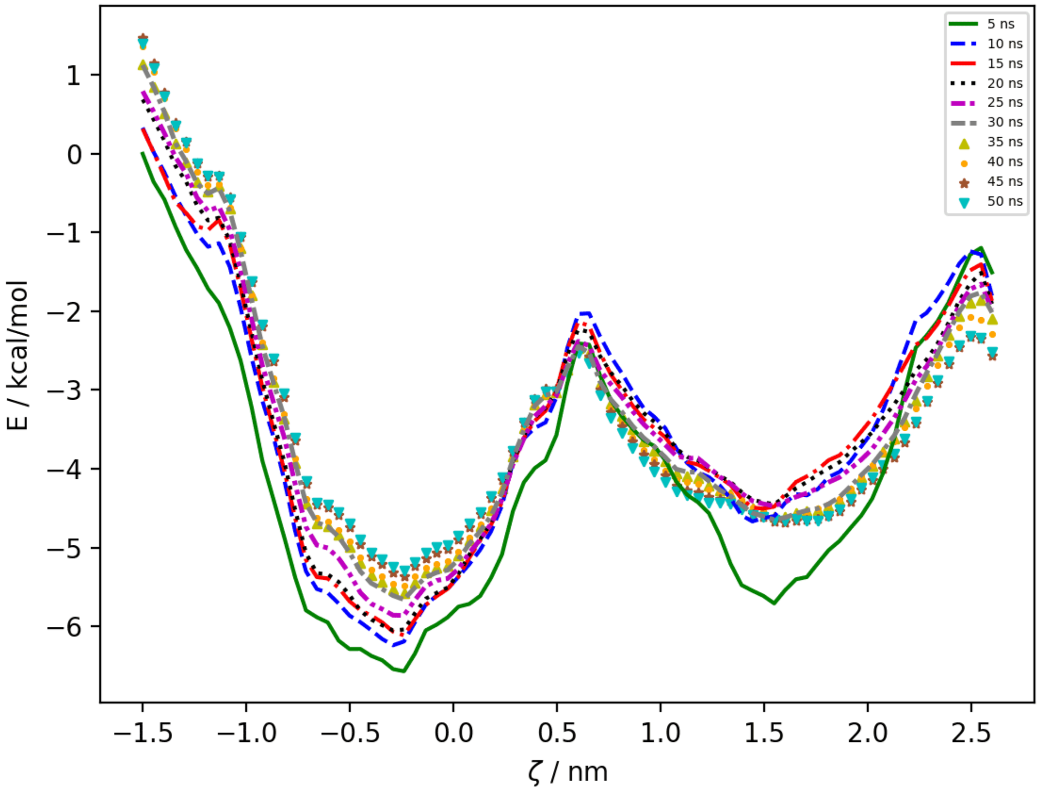

Figure S12. Convergence of the PMF for cuc estimated over increasing simulation times (5 ns equals 0-5 ns of the individual simulations, 50 ns equals the entire simulation time of 50 ns of all individual trajectories). The curves were optimally aligned by calculating their root mean sqare deviation from each other in Python: $\sqrt{\langle \overline{PMF1 - PMF2^2} \rangle}$

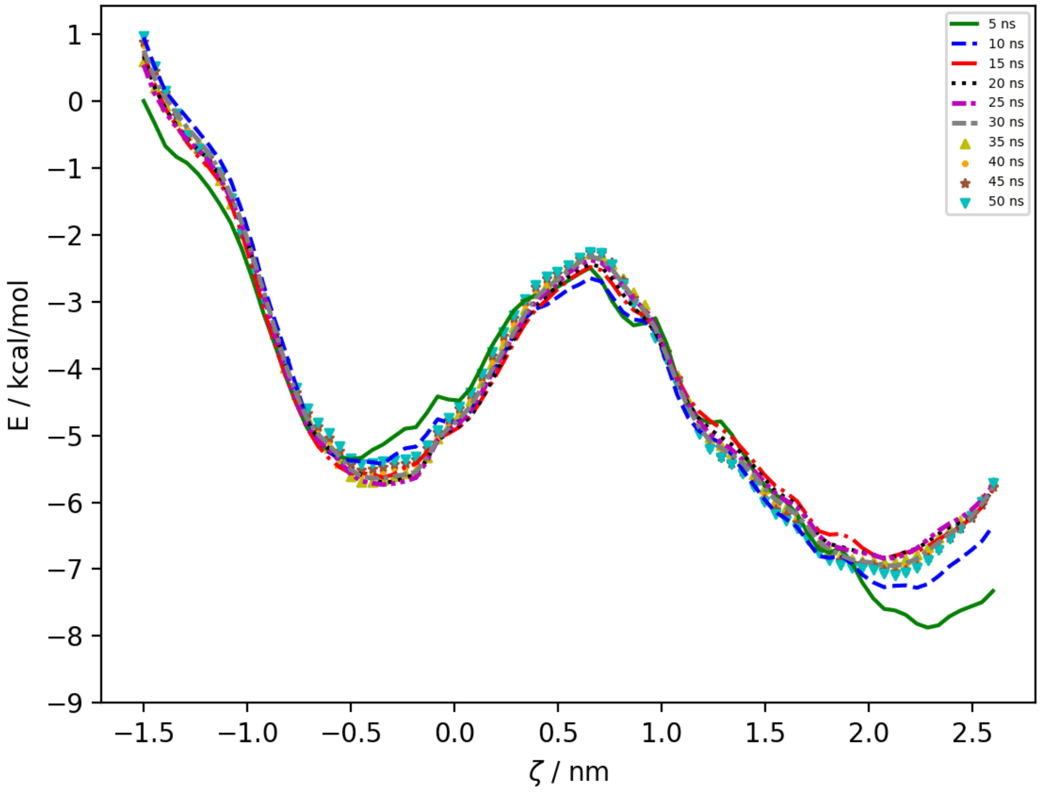

Figure S13. Convergence of the PMF for ucu estimated over increasing simulation times (5 ns equals 0-5 ns of the individual simulations, 50 ns equals the entire simulation time of 50 ns of all individual trajectories). The curves were optimally aligned by calculating their root mean sqare deviation from each other in Python: $\sqrt{\langle PMF1 - PMF2^2 \rangle}$

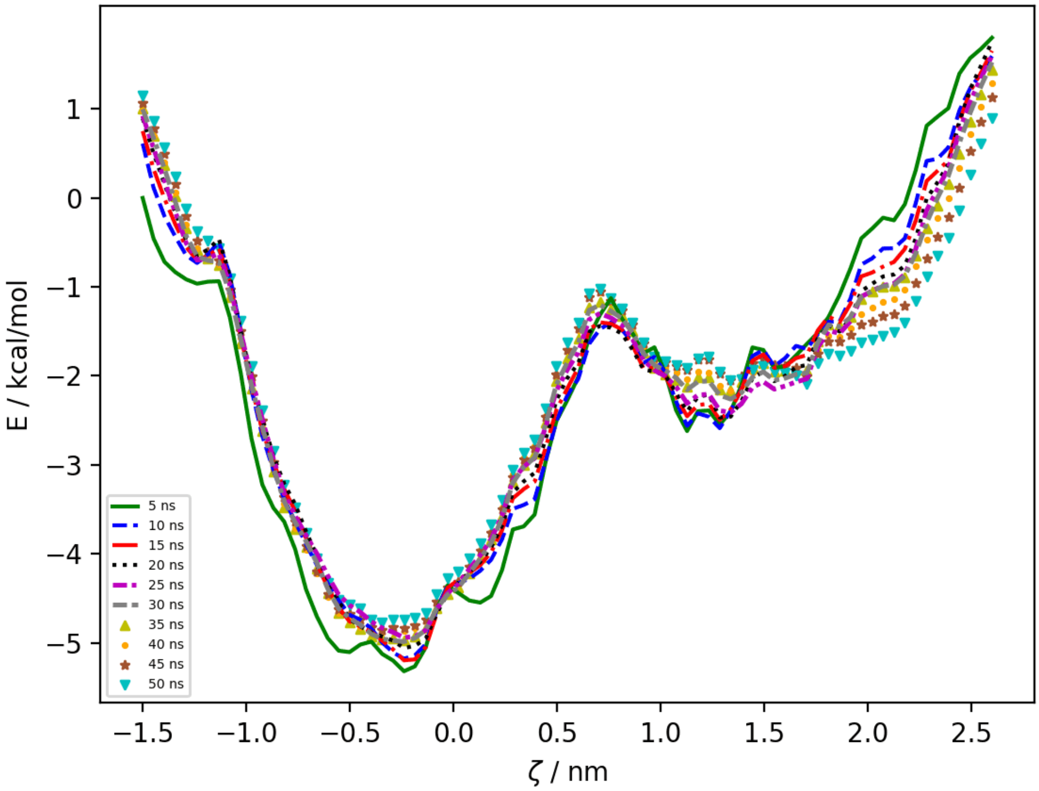

Figure S14. Convergence of the PMF for uuu estimated over increasing simulation times (5 ns equals 0-5 ns of the individual simulations, 50 ns equals the entire simulation time of 50 ns of all individual trajectories). The curves were optimally aligned by calculating their root mean sqare deviation from each other in Python: $\sqrt{\langle PMF1 - PMF2^2 \rangle}$

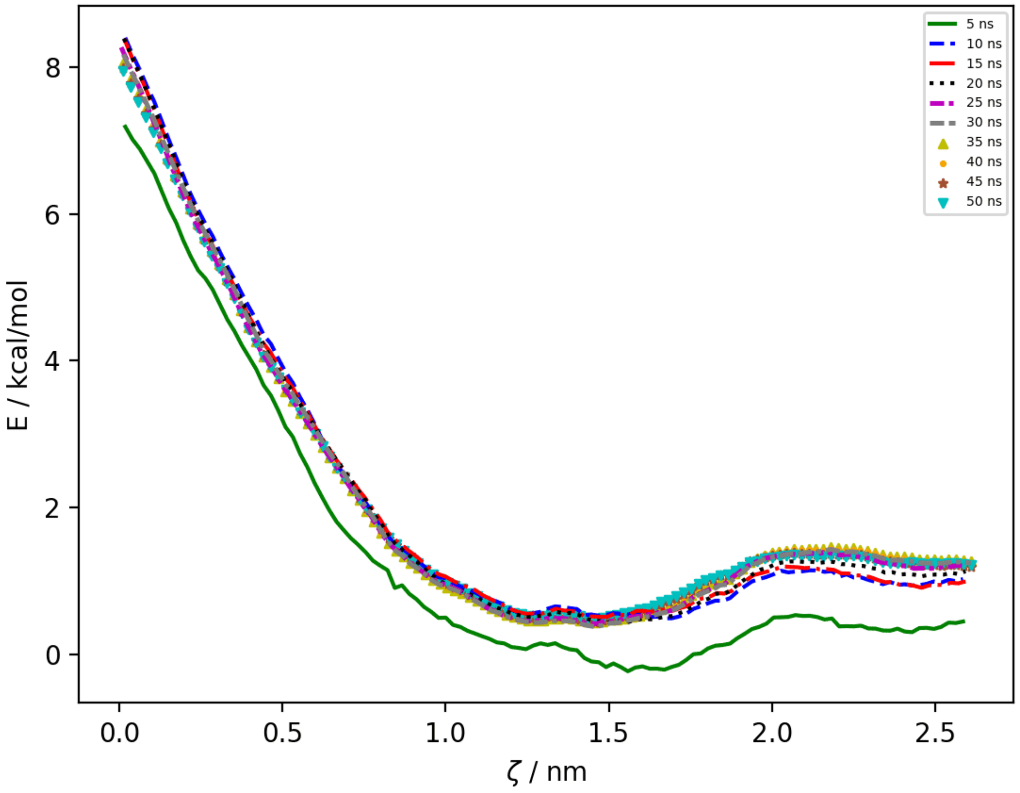

Figure S15. Convergence of the PMF of the charged lysine analog estimated over increasing simulation times (5 ns equals 0-5 ns of the individual simulations, 50 ns equals the entire simulation time of 50 ns of all individual trajectories). The curves were optimally aligned by calculating their root mean sqare deviation from each other in Python: $\sqrt{\langle PMF1 - PMF2^2 \rangle}$

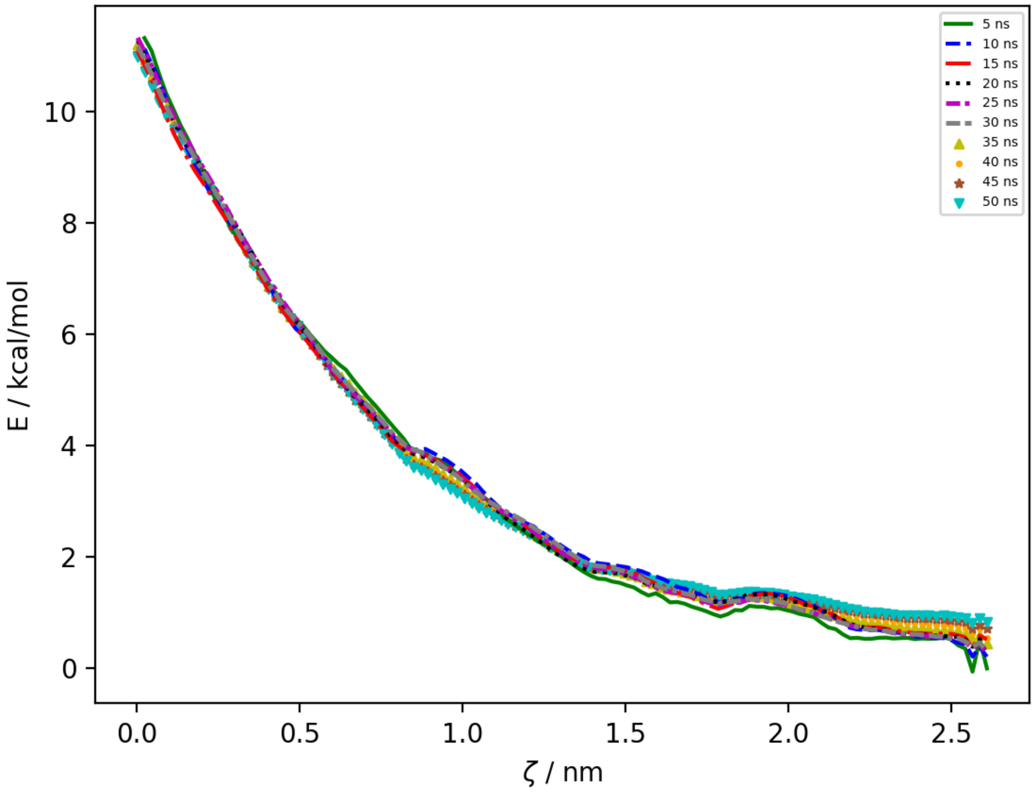

Figure S16. Convergence of the PMF of the charged lysine estimated over increasing simulation times (5 ns equals 0-5 ns of the individual simulations, 50 ns equals the entire simulation time of 50 ns of all individual trajectories). The curves were optimally aligned by calculating their root mean sqare deviation from each other in Python: $\sqrt{\langle \overline{PMF1 - PMF2^2} \rangle}$

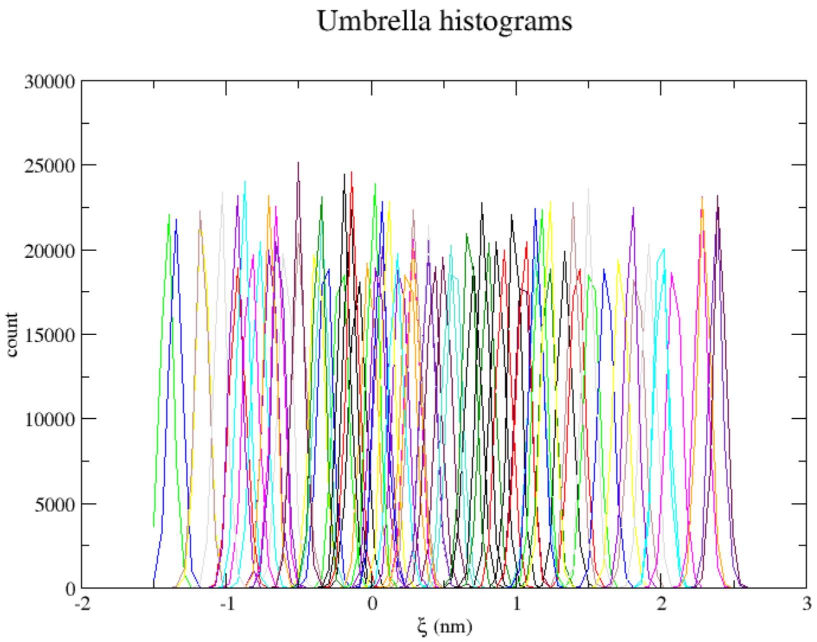

Figure S17. Histograms of the PMF for the ccc charge state.

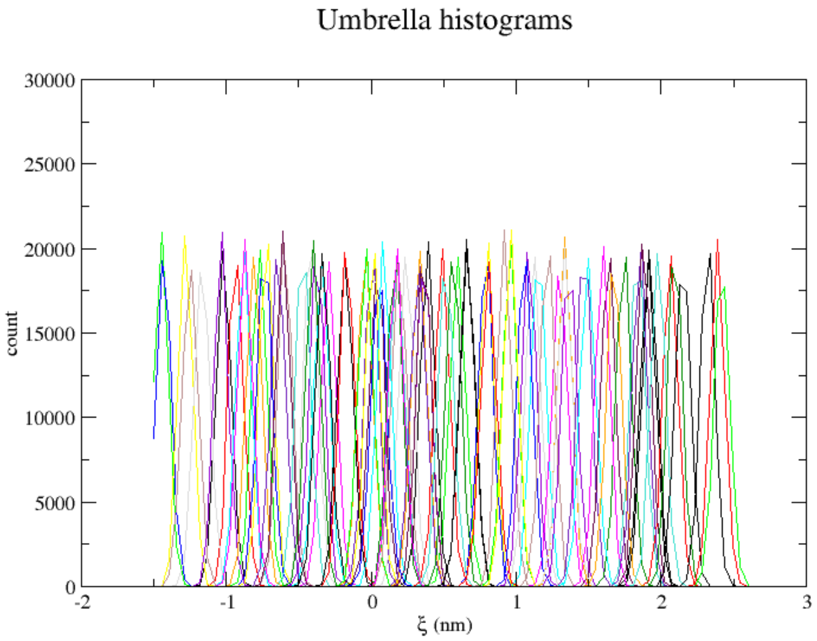

Figure S18. Histograms of the PMF for the cuc charge state.

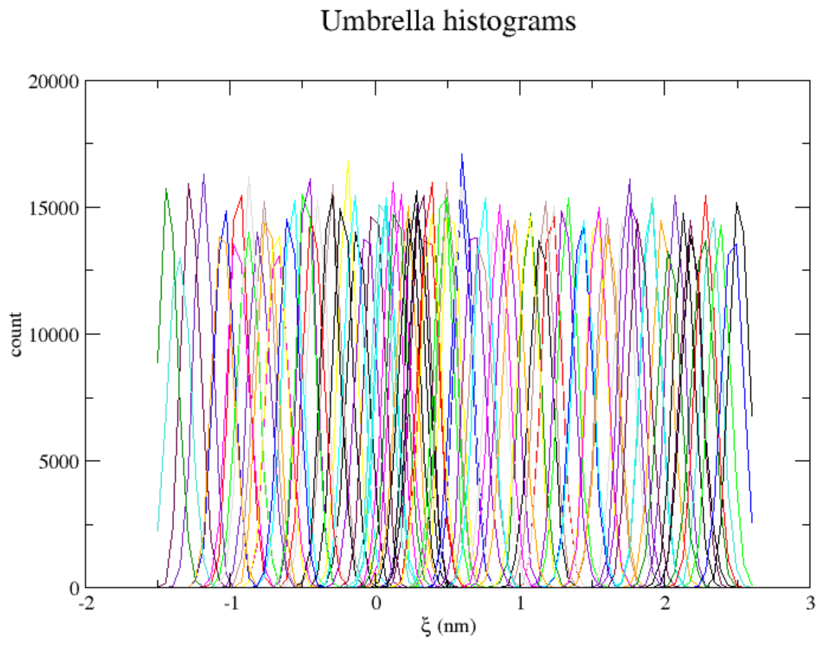

Figure S19. Histograms of the PMF for the ucu charge state.

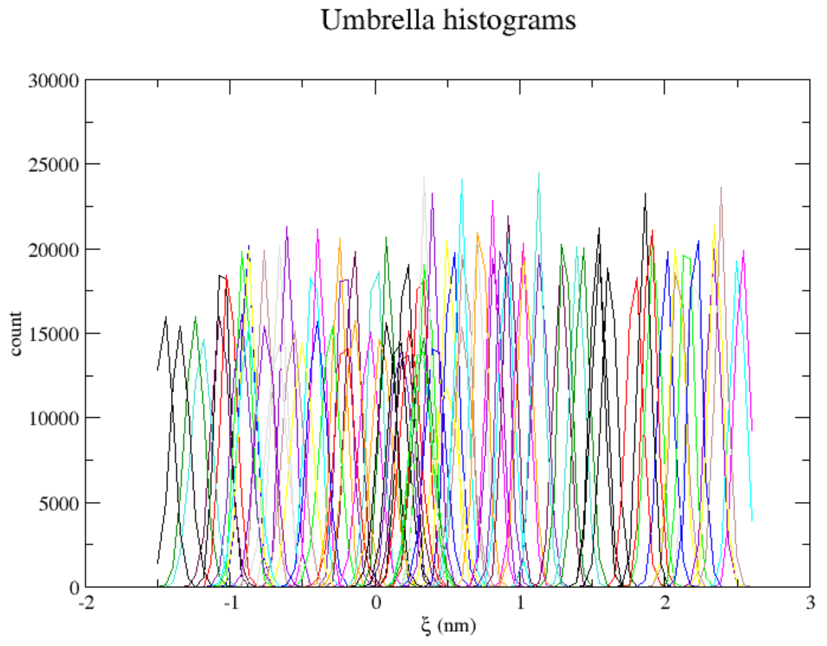

Figure S20. Histograms of the PMF for the uuu charge state.

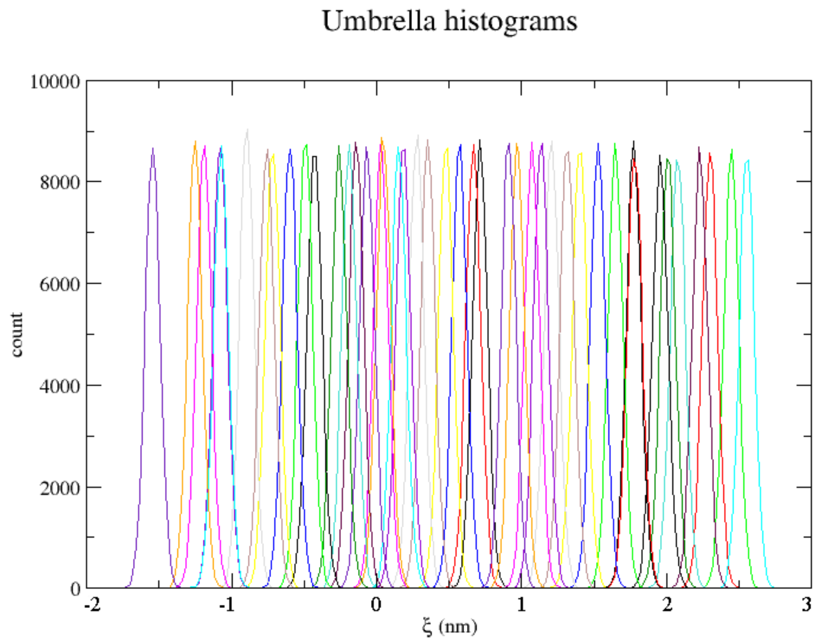

Figure S21. Histograms of the PMF of the charged lysine analog.

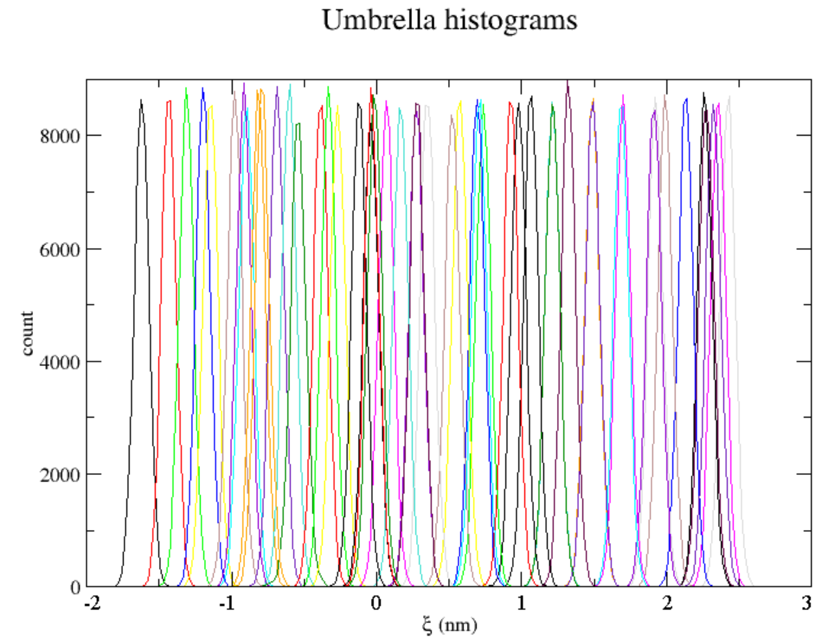

Figure S22. Histograms of the PMF of the charged lysine.

2.4 Contacts of lysines with lipid headgroups

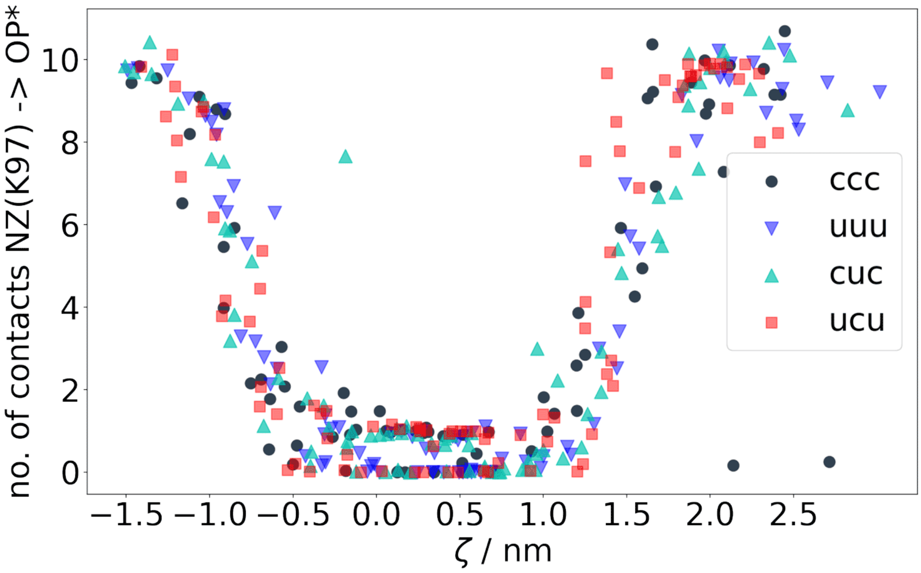

Figure S23. Number of contacts of K97 with phosphate headgroups of lipids averaged over each entire trajectory from the geometric perturbation.

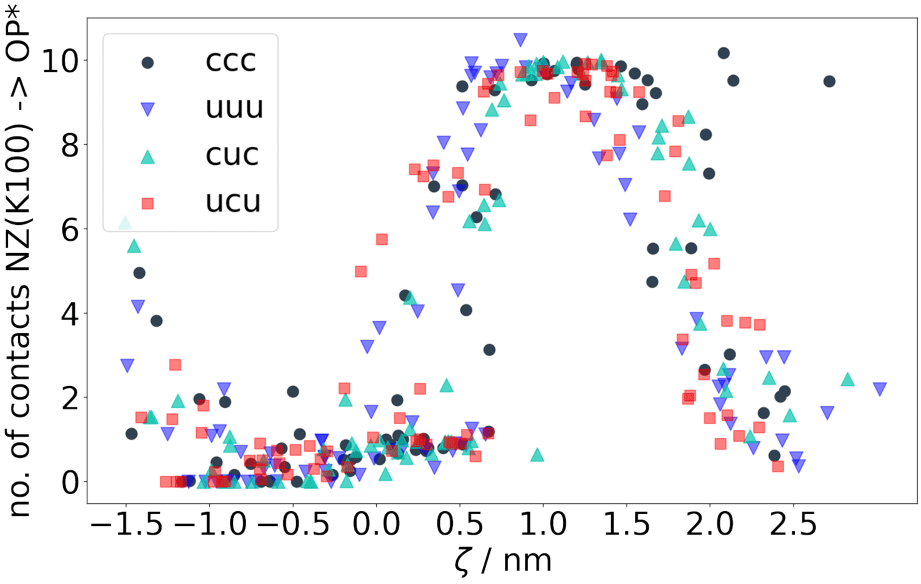

Figure S24. Number of contacts of K100 with phosphate headgroups of lipids averaged over each entire trajectory from the geometric perturbation.

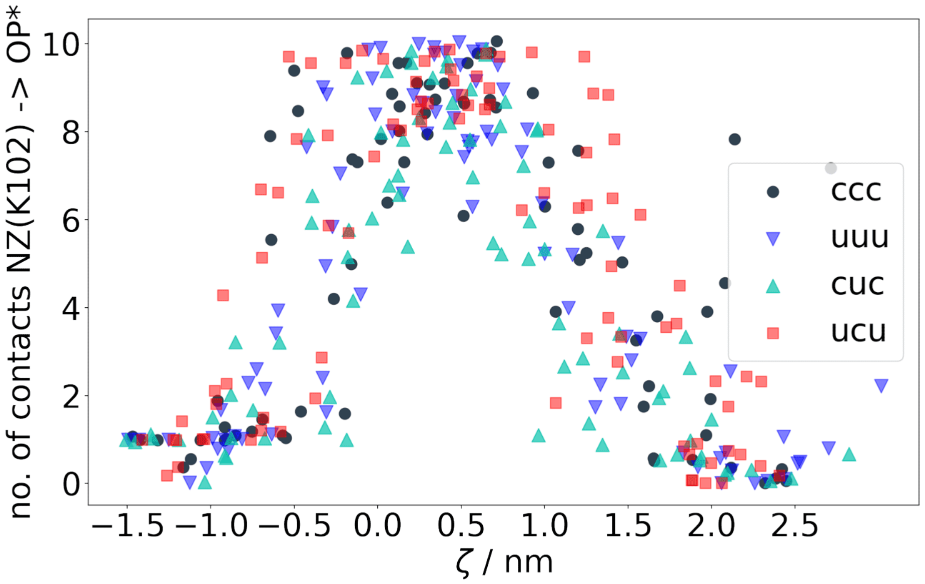

Figure S25. Number of contacts of K102 with phosphate headgroups of lipids averaged over each entire trajectory from the geometric perturbation.

2.5 Water defects of lysine and its analog

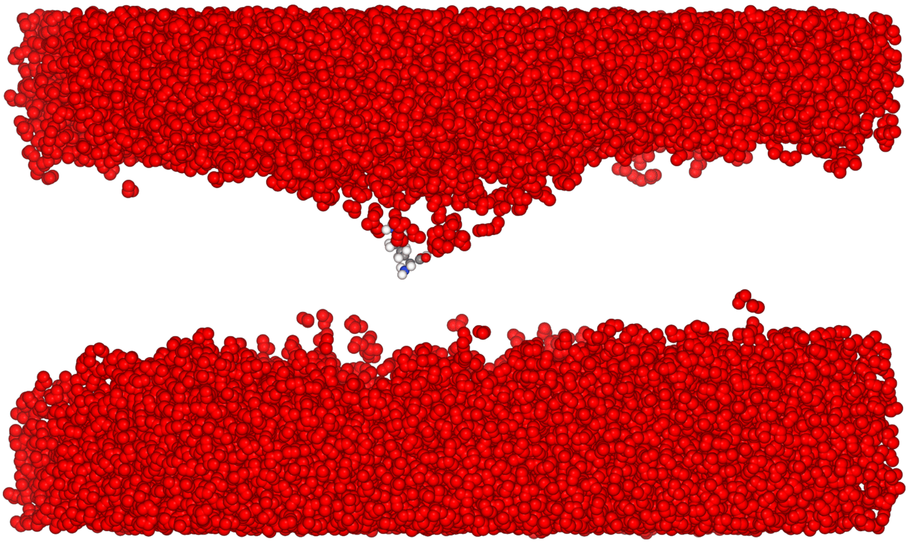

Figure S26. Water defect formed by lysine at $\zeta = 0.05$ nm. Corresponds to a defect containing 18 water molcules at 27.3 ns.

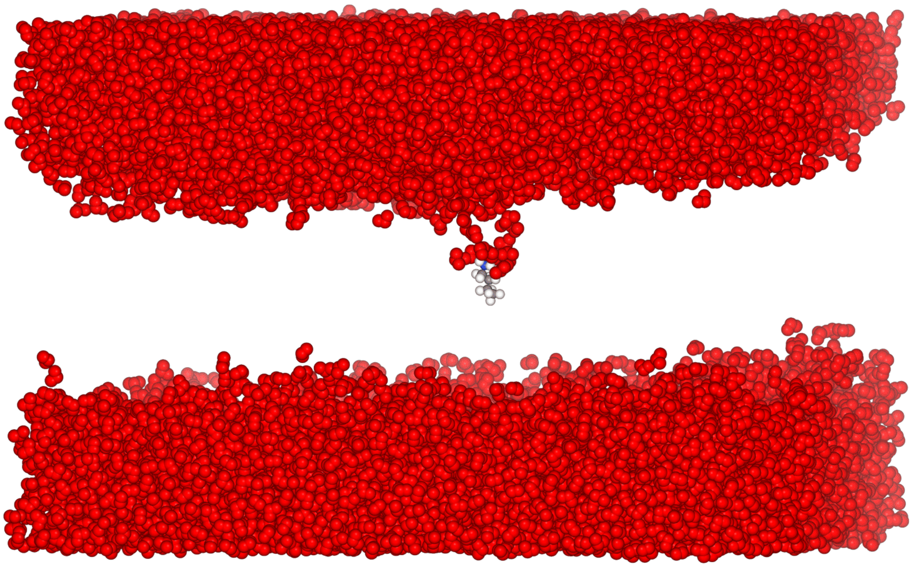

Figure S27. Water defect formed by the lysine analog at $\zeta = 0.03$ nm. Corresponds to a defect containing 9 water molcules at 8.95 ns.

2.6 7-mer arc

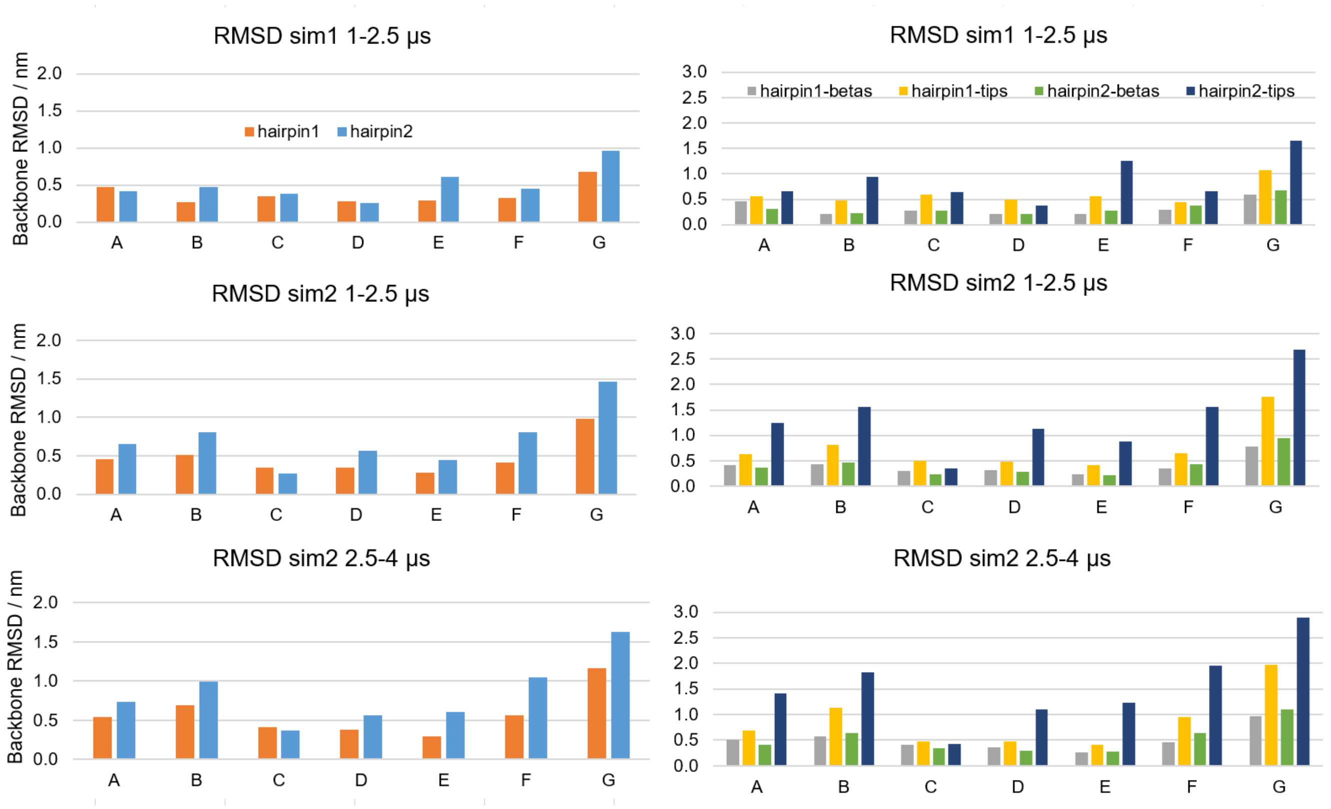

Figure S28. RMSD of the 7-mer arc simulations. hairpin1 = residues 80-113, hairpin2 = residues 164-198, hairpin1-tips = residues 95-99, hairpin1-betas = 80-94 and 100-113, hairpin2-tips = residues 178-184, and hairpin2-betas = residues 164-177 and 185-198. The seven capital letters correspond to the seven chains.

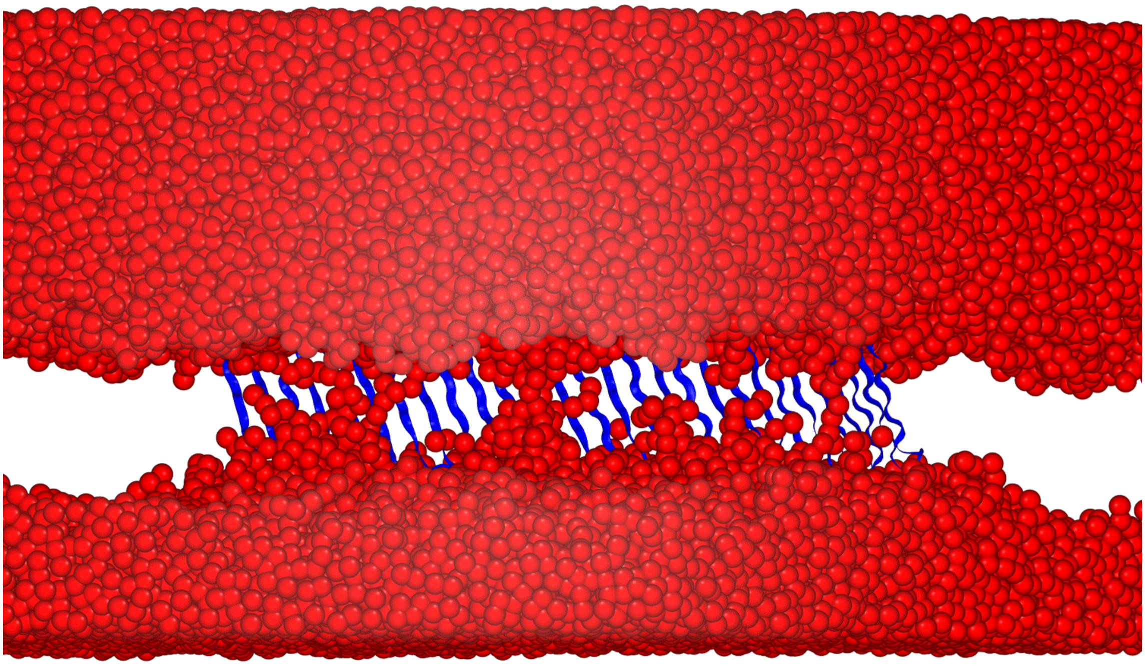

Figure S29. Water defects present at the beginning of the 4µs simulation with the protomers restrained in the pore conformation. This frame shows a defect containing 346 water molecules.

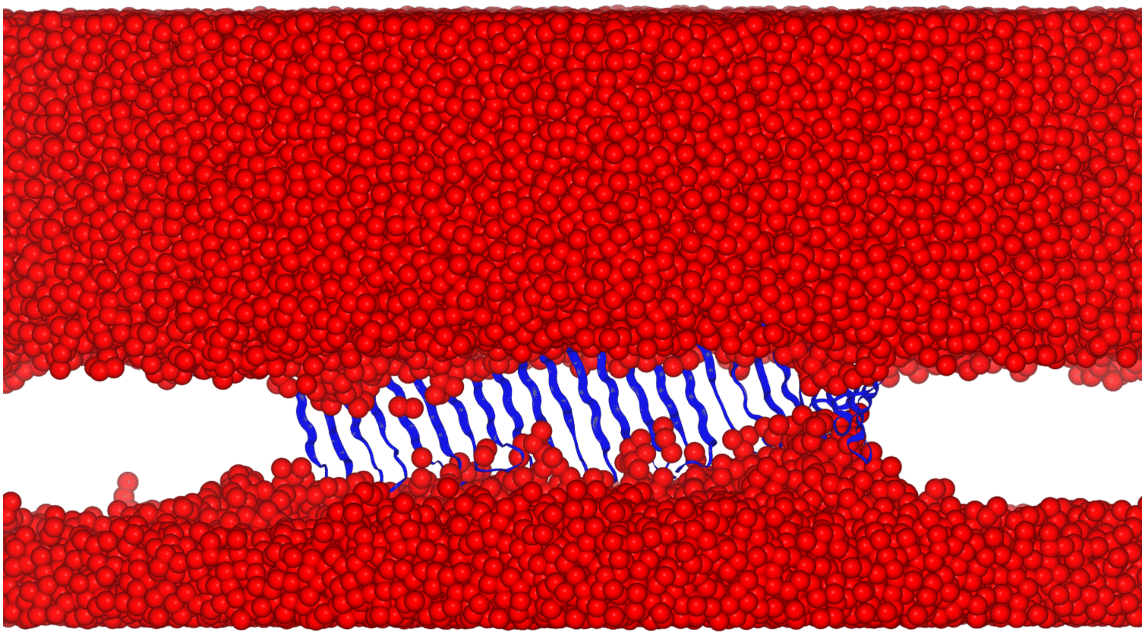

Figure S30. Reduced water defects after 4µs with the relaxed 7-mer arc protomers. This frame shows a defect containing 268 water molecules.

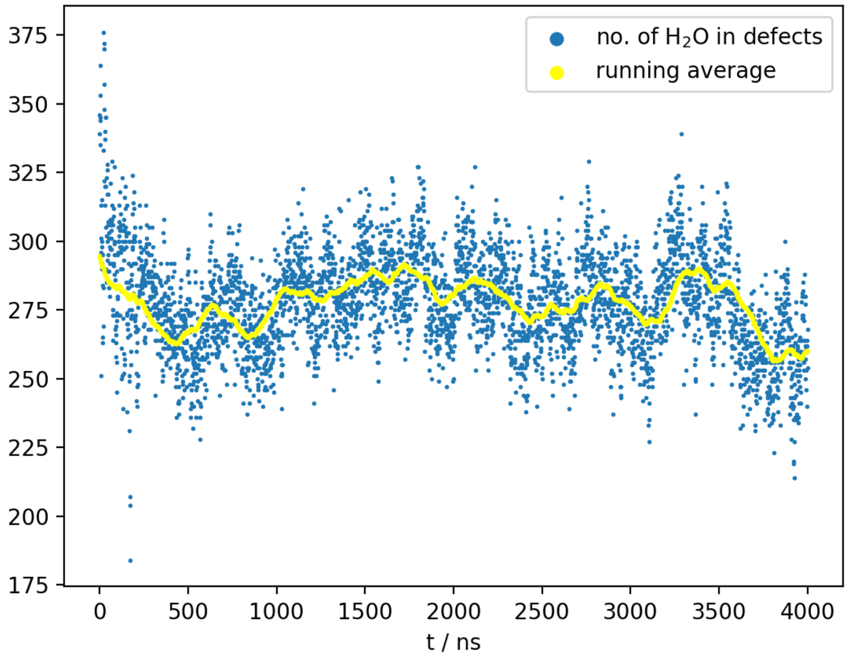

Figure S31. Water defects in the membrane caused by the GSDMA3 7-mer arc over time.

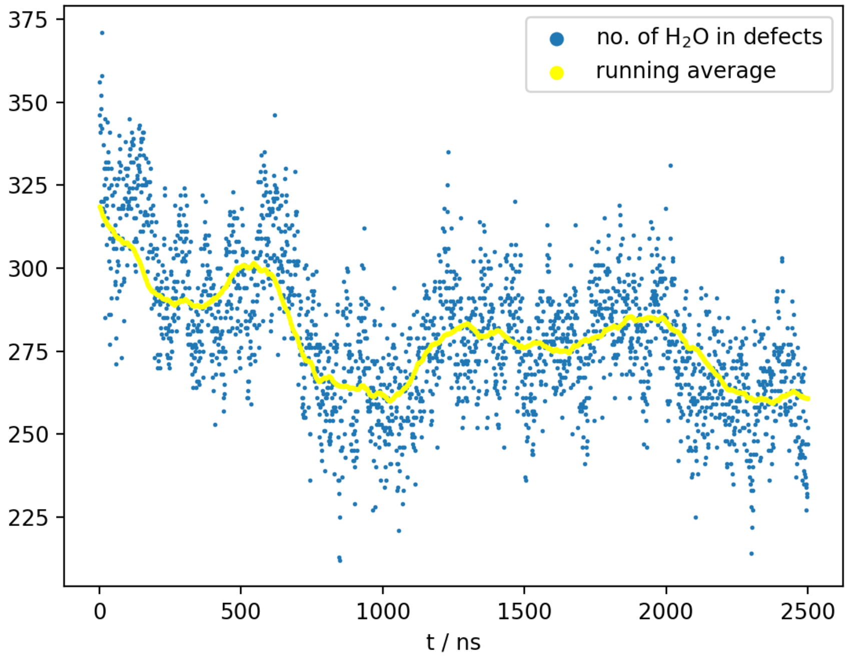

Figure S32. Water defects in the membrane caused by the GSDMA3 7-mer arc over time.

REFERENCES

Abraham, M. J., Murtola, T., Schulz, R., Páll, S., Smith, J. C., Hess, B., et al. (2015). Gromacs: High performance molecular simulations through multi-level parallelism from laptops to supercomputers. *SoftwareX* 1, 19–25

AlphaFold, Gasdermin-C AlphaFold protein structure database (2022), _https://alphafold.ebi.ac.uk/entry/P85967, (accessed 2022-04-14)

Berendsen, H. J., Postma, J. v., van Gunsteren, W. F., DiNola, A., and Haak, J. R. (1984). Molecular dynamics with coupling to an external bath. *The Journal of chemical physics* 81, 3684–3690

Bienert, S., Waterhouse, A., de Beer, T. A., Tauriello, G., Studer, G., Bordoli, L., et al. (2017). The swiss-model repository new features and functionality. *Nucleic acids research* 45, D313–D319

Darden, T., York, D., and Pedersen, L. (1993). Particle mesh Ewald: An N [center-dot] log(N) method for Ewald sums in large systems. *The Journal of Chemical Physics* 98, 10089–10092. doi:_10.1063/1.464397

De Jong, D. H., Baoukina, S., Ingólfsson, H. I., and Marrink, S. J. (2016). Martini straight: Boosting performance using a shorter cutoff and gpus. *Computer Physics Communications* 199, 1–7

Ding, J., Wang, K., Liu, W., She, Y., Sun, Q., Shi, J., et al. (2016). Pore-forming activity and structural autoinhibition of the gasdermin family. *Nature* 535, 111–116

Evans, D. J. and Holian, B. L. (1985). The nose–hoover thermostat. *The Journal of chemical physics* 83, 4069–4074

Gowers, R. J., Linke, M., Barnoud, J., Reddy, T. J. E., Melo, M. N., Seyler, S. L., et al. (2019). *MDAnalysis: a Python package for the rapid analysis of molecular dynamics simulations*. Tech. rep., Los Alamos National Lab.(LANL), Los Alamos, NM (United States)

Guex, N., Peitsch, M. C., and Schwede, T. (2009). Automated comparative protein structure modeling with swiss-model and swiss-pdbviewer: A historical perspective. *Electrophoresis* 30, S162–S173

Hess, B. (2008). P-lincs: A parallel linear constraint solver for molecular simulation. *Journal of chemical theory and computation* 4, 116–122

Huang, J., Rauscher, S., Nawrocki, G., Ran, T., Feig, M., De Groot, B. L., et al. (2017). Charmm36m: an improved force field for folded and intrinsically disordered proteins. *Nature methods* 14, 71–73

Hub, J. S., De Groot, B. L., and Van Der Spoel, D. (2010). g_wham? a free weighted histogram analysis implementation including robust error and autocorrelation estimates. *Journal of chemical theory and computation* 6, 3713–3720

Hunt, I., Table of pKa and pI values (2022), _https://www.chem.ucalgary.ca/courses/351/Carey5th/Ch27/ch27-1-4-2.html, (accessed 2022-04-11)

Jorgensen, W. L. and Madura, J. D. (1985). Temperature and size dependence for monte carlo simulations of tip4p water. *Molecular Physics* 56, 1381–1392

Klauda, J. B., Venable, R. M., Freites, J. A., O'Connor, J. W., Tobias, D. J., Mondragon-Ramirez, C., et al. (2010). Update of the charmm all-atom additive force field for lipids: validation on six lipid types. *The journal of physical chemistry B* 114, 7830–7843

Kumar, S., Rosenberg, J. M., Bouzida, D., Swendsen, R. H., and Kollman, P. A. (1992). The weighted histogram analysis method for free-energy calculations on biomolecules. i. the method. *Journal of computational chemistry* 13, 1011–1021

Mari, S. A., Pluhackova, K., Pipercevic, J., Leipner, M., Hiller, S., Engel, A., et al. (2022). Gasdermin-A3 pore formation propagates along variable pathways. *Nat. Commun.* 13, 2609

Michaud-Agrawal, N., Denning, E. J., Woolf, T. B., and Beckstein, O. (2011). Mdanalysis: a toolkit for the analysis of molecular dynamics simulations. *Journal of computational chemistry* 32, 2319–2327

O'Neil, M. e. (2006). *The Merck Index - An Encyclopedia of Chemicals, Drugs, and Biologicals* (Whitehouse Station, NJ: Merck and Co., Inc.)

Páll, S. and Hess, B. (2013). A flexible algorithm for calculating pair interactions on simd architectures. *Computer Physics Communications* 184, 2641–2650

Parrinello, M. and Rahman, A. (1980). Crystal structure and pair potentials: A molecular-dynamics study. *Physical review letters* 45, 1196

Parrinello, M. and Rahman, A. (1981). Polymorphic transitions in single crystals: A new molecular dynamics method. *Journal of Applied physics* 52, 7182–7190

Pluhackova, K. and Horner, A. (2021). Native-like membrane models of e. coli polar lipid extract shed light on the importance of lipid composition complexity. *BMC biology* 19, 1–22

Python, Python software foundation. python language reference, version 3. available at `http://www.python.org` (accessed 2020-06-08) (2020)

Ruan, J., Xia, S., Liu, X., Lieberman, J., and Wu, H. (2018). Cryo-em structure of the gasdermin a3 membrane pore. *Nature* 557, 62–67

Souaille, M. and Roux, B. (2001). Extension to the weighted histogram analysis method: combining umbrella sampling with free energy calculations. *Computer physics communications* 135, 40–57

Souza, P., Alessandri, R., and Barnoud, J. e. a. (2021). Martini 3: a general purpose force field for coarse-grained molecular dynamics. *Nat Methods* 18, 382–388

Studer, G., Rempfer, C., Waterhouse, A. M., Gumienny, R., Haas, J., and Schwede, T. (2020). Qmeandisco distance constraints applied on model quality estimation. *Bioinformatics* 36, 1765–1771

Wassenaar, T. A., Ingólfsson, H. I., Böckmann, R. A., Tieleman, D. P., and Marrink, S. J. (2015a). Computational lipidomics with insane: a versatile tool for generating custom membranes for molecular simulations. *Journal of chemical theory and computation* 11, 2144–2155

Wassenaar, T. A., Pluhackova, K., Böckmann, R. A., Marrink, S. J., and Tieleman, D. P. (2014). Going backward: a flexible geometric approach to reverse transformation from coarse grained to atomistic models. *Journal of chemical theory and computation* 10, 676–690

Wassenaar, T. A., Pluhackova, K., Moussatova, A., Sengupta, D., Marrink, S. J., Tieleman, D. P., et al. (2015b). High-throughput simulations of dimer and trimer assembly of membrane proteins. the daft approach. *Journal of chemical theory and computation* 11, 2278–2291

Waterhouse, A., Bertoni, M., Bienert, S., Studer, G., Tauriello, G., Gumienny, R., et al. (2018). Swiss-model: homology modelling of protein structures and complexes. *Nucleic acids research* 46, W296–W303

Webb, B. and Sali, A. (2016). Comparative protein structure modeling using modeller. *Current protocols in bioinformatics* 54, 5–6

Xia, S., Zhang, Z., Magupalli, V. G., Pablo, J. L., Dong, Y., Vora, S. M., et al. (2021). Gasdermin d pore structure reveals preferential release of mature interleukin-1. *Nature* 593, 607–611
